# Effects of biological and abiotic factors on dark septate endophytes distribution and heavy metal resistance in different environments

**DOI:** 10.3389/fmicb.2024.1527512

**Published:** 2025-01-10

**Authors:** Zhenzhou Wang, Wenyi Shi, Xiuling Chen, Yuli Lin, Wenjing Chen, Li Yao, Xiang Sun, Xia Li, Xueli He

**Affiliations:** ^1^School of Life Sciences, Hebei University, Baoding, China; ^2^Key Laboratory of Microbial Diversity Research and Application of Hebei Province, Baoding, China; ^3^Engineering Research Center of Ecological Safety and Conservation in Beijing-Tianjin-Hebei (Xiong’an New Area) of MOE, Baoding, China

**Keywords:** dark septate endophytes, distribution, fungal diversity, heavy metal habitats, heavy metal resistance

## Abstract

**Introduction:**

Exploring the interactions between dark septate endophytes (DSE) in plant roots across diverse heavy metal habitats—considering host plants, site characteristics, and microbial communities—provides insights into the distribution patterns of DSE in metal-rich environments and their mechanisms for developing heavy metal resistance.

**Methods:**

This study collected samples of three common plant species (*Phragmites australis*, PA, *Setaria viridis*, SV, and *Artemisia annua*, AA) and their corresponding soil samples from three heavy metal-contaminated sites: Baiyang Lake, BY, Fengfeng mining area, FF, and Huangdao, HD. Utilizing high-throughput sequencing and physicochemical analysis methods, the biological and abiotic factors affecting DSE colonization and distribution in the roots were investigated.

**Results:**

Twenty-two DSE species were isolated and identified with 11 species found in BY, 8 species in FF, and 11 species in HD. The screening for heavy metal resistance discovered 10 heavy metal-tolerant DSE species. Soil available phosphate, available nitrogen, and Cd contents, as well as the composition of different root fungal communities, influence the resistance and distribution of heavy metal-tolerant DSE. Notably, 12 DSE species, including *Paraphoma radicina* and *Paraphoma chrysanthemicola*, were reported for the first time in heavy metal habitats. The colonization rates of DSE in the roots of PA (96%) and AA (76%) were highest in BY, while the highest colonization rate in the roots of SV was observed in HD (94%). Site-specific soil parameters, such as available K, organic contents, Zn, and Cd contents from different sites are the main determinants affecting DSE colonization. Meanwhile, the diversity and richness of other root-associated endophytic fungi, which varied considerably across sites, served as significant biological factors influencing DSE distribution and colonization.

**Discussion:**

The results of this study provide a strong theoretical framework for the effective utilization of DSE fungi to mitigate soil heavy metal pollution.

## Graphical abstract

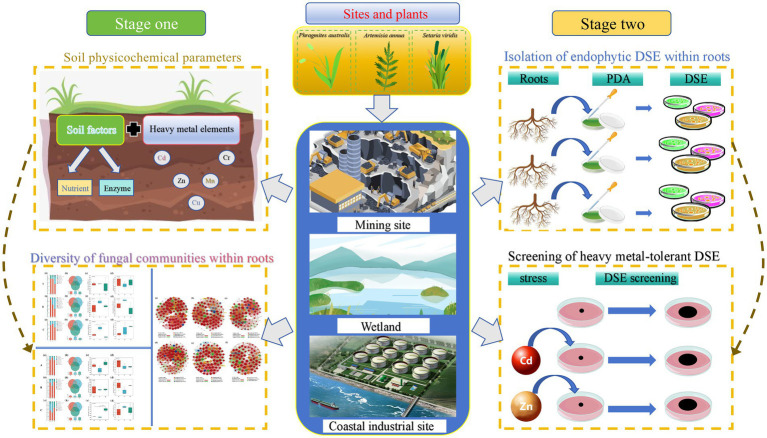

## 1 Introduction

With rapid global industrialization, heavy metal pollution has become a pressing global concern (Zheng et al., 2023). Due to their high toxicity, non-degradability, and accumulation within organisms, heavy metals pose persistent threats to environmental and human health (Duan et al., 2021; Li et al., 2022b). In plants, heavy metals adversely impact cellular and molecular functions by inactivating enzymes, denaturing proteins, disrupting the metabolism of essential molecules, displacing necessary metal ions from biomolecules, and compromising membrane integrity (Yaashikaa et al., 2022). Consequently, effective heavy metal remediation has become a priority in environmental management and sustainable development (Yan et al., 2022). However, physical and chemical remediation approaches are often limited by high costs and inefficiency (Patel et al., 2022). In recent years, phytoremediation has gained attention for its potential to mitigate heavy metal contamination, as plants can accumulate and translocate heavy metals through their root systems. Nonetheless, the reduced nutrient availability and elevated heavy metal concentrations in polluted soils inhibit root growth, limiting the effectiveness of phytoremediation. In this context, dark septate endophytes (DSE) can form mutualistic relationships with plants, enhancing host survival and promoting heavy metal uptake. Additionally, DSE cell walls have the capacity to adsorb heavy metals, further facilitating the remediation process (Jeyakumar et al., 2022).

DSE fungi represent an important group of root-associated symbionts characterized by pigmented septate hyphae and microsclerotia within plant roots (Jumpponen and Trappe, 1998). Studies have shown that DSE are widely distributed across diverse habitats worldwide. To date, 136 genera comprising 196 DSE species have been isolated from the roots of various plants—including *Phragmites australis*, *Oryza granulata*, *Setaria viridis*, and *Salvia miltiorrhiza*—inhabiting environments with heavy metals, drought, wetlands, salinity, and tropical conditions (Hou et al., 2020; Li et al., 2024; Xu et al., 2023). DSE are particularly prominent in these stress-prone habitats. For example, under high salinity, DSE colonization increases with salt concentration, alleviating host salt stress by raising osmotic pressure and producing trehalose and mannitol. In drought environments, DSE colonization enhance plant drought resistance by boosting antioxidant activity and regulating osmotic balance (Li et al., 2022a; Mateu et al., 2020). These findings underscore the critical role of DSE in supporting plant survival under challenging conditions, such as heavy metal toxicity, salinity, and drought stress.

DSE has demonstrated effectiveness in mitigating heavy metal pollution through various mechanisms. For instance, Su et al. (2021) reported that *Falciphora oryzae*, a DSE isolated from *Oryza granulata*, effectively reduces cadmium (Cd) content in rice by employing metal sequestration and chelation systems, thereby promoting plant growth. Similarly, Wang et al. (2023) showed that inoculating maize with DSE reduced Cd solubility and inorganic content, increased plant growth regulator levels, and lowered abscisic acid (ABA) levels, resulting in enhanced growth and reduced Cd toxicity. These studies highlight DSE’s capacity to improve plant growth under metal stress, underscoring its potential role in phytoremediation strategies. However, conclusions derived from controlled inoculation studies may not fully reflect the complexity of plant-fungal mutualisms in natural ecosystems. Our understanding of the distribution patterns and diversity of DSE in natural settings remains limited, which restricts the effective utilization of DSE in environmental applications. In natural environments, DSE distribution often varies according to habitat conditions. For example, Li et al. (2024) reported significant differences in DSE colonization across desert sites with varying properties, suggesting that DSE may exhibit habitat preferences. A comprehensive understanding of these distribution patterns in natural ecosystems remains a key focus of DSE research.

The colonization of DSE is influenced by plant species and soil environments (Hou et al., 2019; Sudová et al., 2020). For the biological factors, Han et al. (2021) assessed the DSE colonization rates in 25 medicinal plants and found a wide range of colonization, from 2.2 to 100%, with *Taraxacum mongolicum* in the Asteraceae family showing the highest rate (100%). In stressed environments, such as deserts and arid regions, soil factors like pH and organic matter content significantly impact DSE colonization rates across sampling sites (Li et al., 2022b; Zuo et al., 2022). Similarly, in heavy metal-contaminated environments, plant identity and environmental factors shape DSE colonization. For instance, Xu et al. (2023) reported that soil pH and Cd content significantly influenced DSE colonization across various sites, while Becerra et al. (2023) found that DSE colonization in Pb-polluted areas was strongly affected by soil metal concentrations, plant species traits, and plant metal concentrations. The levels of heavy metals and other abiotic factors, such as soil nutrients, significantly influence the colonization of fungi like DSE. As heavy metal concentrations in soil increase, microbial biomass carbon decreases correspondingly. Heavy metals, including Cd, Zn, and Cu, can disrupt cell structure and function, accelerate cell death, and inhibit microbial activity or competitiveness, thereby affecting fungal colonization (Tang et al., 2021). The colonization of DSE in the host relies heavily on effective carbon sources from the soil. Organic carbon serves as the primary carbon source for microbial communities, and the rate of organic matter decomposition plays a crucial role in shaping DSE colonization within the host (Han et al., 2021). Therefore, identifying the key factors influencing DSE colonization is essential to optimize DSE application in phytoremediation efforts.

Beyond plant species, interactions with other members of the endophytic fungal community in the roots, may also influence DSE colonization success. Within the root community, endophytes may exhibit mutualistic or antagonistic interactions (Xu et al., 2020). Fungal communities inhabit diverse ecological niches and engage in complex interactions, competing for nutrients and space with other fungi at both interspecific and intraspecific levels. These interactions involve mechanisms such as competitive growth, antibiotic competition, and direct parasitism of hosts at various life stages. Additionally, some fungal species acquire nutrients by preying on other fungi, insects, nematodes, and their surviving structures. Endophytic fungi like DSE contribute to community formation by fostering synergistic interactions (Piombo et al., 2024; Tao et al., 2023). When DSE successfully colonize host roots, secretions from other root-associated fungi can impact the mutualistic relationship between DSE and the host plant, affecting both colonization intensity and the production of growth regulators like auxin by DSE (Alzarhani et al., 2019). Consequently, investigating the co-occurrence of DSE with other endophytic fungi and their interspecies interactions is crucial for a more comprehensive understanding of DSE distribution and colonization patterns within root communities.

Although previous studies have demonstrated that DSE distribution is influenced by multiple factors, most research has focused on either biological or single abiotic factors (Sudová et al., 2020). To explore the combined influence of multiple factors—including plant species, soil properties, and other endophytes—on DSE colonization and distribution, we conducted a study across three heavy metal-contaminated sites: Baiyang Lake wetland (BY), Fengfeng mining area (FF), and Huangdao coastal industrial district (HD) in Qingdao city (Wang et al., 2024a; Zhang et al., 2024; Zheng et al., 2023). These sites contain stress-tolerant plants—*Artemisia annua* (AA), *Phragmites australis* (PA), and *Setaria viridis* (SV)—that are widely distributed in each location (Nong et al., 2022; Tripathi et al., 2020; Xu et al., 2023). Our investigation focused on the distribution, species diversity, and soil properties associated with DSE across these plant species. High-throughput sequencing was employed to analyze the species composition of the entire root endophyte community, followed by screening isolated DSE for heavy metal resistance. We proposed the following hypotheses: (1) There are significant differences in the colonization and isolation of DSE in various heavy metal areas and among different plants; (2) the soil properties and endophytic fungal communities in different heavy metal sites are the main factors affecting DSE distribution.

## 2 Materials and methods

### 2.1 Collection of plant and soil samples

Samples were collected from three locations in northern China in July 2023. Baiyang Lake (BY, 38° 43′ N, 115° 45′ E) is situated near Baoding City, Hebei Province, China. This lake, the largest wetland in northern China with an area of 366 km^2^, has recently faced threats from eutrophication and cadmium pollution. The Fengfeng mining area (FF, 36° 20’ N, 114° 3′ E) in Handan City, Hebei Province, is a coal and iron ore production site affected by multiple heavy metals, including Cr, Cd, and Cu (Song et al., 2023). The Huangdao coastal industrial site (HD, 35° 46′ N, 119° 54′ E) in Qingdao City, Shandong Province, has experienced significant biodiversity loss and soil contamination from heavy metals and organic pollutants due to oil spills and industrial expansion (Wang et al., 2024a; Zhang et al., 2024; Zheng et al., 2023). The average annual temperature of Baiyang Lake is 12.2°C, the average annual precipitation is 563.9 mm. The average annual temperature of Fengfeng mining site is 15.7°C, the average annual precipitation is 1206.1 mm. The average annual temperature of Huangdao is 13.9°C, the average annual precipitation is 1213.7 mm. The vegetations in all three sites were dominated with artemi-sinin produced *Artemisia annua* (AA), hyperaccumulator *Phragmites australis* (PA), and drought resistant and heavy metal tolerant plant, *Setaria viridis* (SV) (Nong et al., 2022; Tripathi et al., 2020; Xu et al., 2023). At each site, three plots of 500 × 500 m^2^ were established, with distances of over 5 km between plots. Five replicates of plant and soil samples were collected from a depth of 0–30 cm at each sampling site. The samples were sealed in plastic bags, transported to the laboratory, and immediately stored at −80°C.

### 2.2 Measurement of soil physiochemical parameters

Soil pH was measured using a pH meter 3,000, following a 1:2.5 (v/w) soil-to-distilled water ratio and allowing the mixture to stand. Soil organic matter (OC) content was determined using the ignition method (Heiri et al., 2001). Available phosphorus (AP) was quantified *via* the colorimetric method (Zuo et al., 2022), while available potassium (AK) was determined using the sodium tetraphenylborate method (Chen et al., 2023). Available nitrogen (AN) was assessed to determine soil nitrogen availability, using the alkali hydrolysis diffusion method. Total nitrogen (TN) and total phosphorus (TP) contents were analyzed using a Smartchem 200 analyzer (Alliance, France) (Xie et al., 2017). Soil alkaline phosphatase (ALP) activity was measured using a modified Bremner and Tabatabai method (Tarafdar and Marschner, 1994), while urease (URE) activity was determined with a modified Hoffmann and Teicher colorimetric method (Hoffmann and Teicher, 1961). Heavy metal concentrations (Cr, Zn, Cu, Mn, and Cd) in the soil were measured *via* ICP-MS. For metal quantification, a mixture of 4 mL HNO_3_, 2 mL HCl, and 2 mL HF was used to decompose 0.3 g of each soil sample (Liu et al., 2018). The concentrations of exchangeable heavy metals (Cr, Zn, Cu, Mn, Cd) in soil were determined using the ICP-MS method. For quantitative metal analysis, 8 mL of MgCl₂ solution was used to extract exchangeable heavy metals from 1 g of each soil sample (He et al., 2024).

### 2.3 Quantification of fungal colonization

Fresh root segments (0.5 cm) were excised and rinsed with sterile water to remove any external soil particles and impurities. After heat treatment in a 10% (w/v) KOH solution at 100°C for 1 h, the samples were thoroughly rinsed with distilled water until the root segments became translucent. The samples were then stained with a 0.5% (w/v) acid fuchsin solution for 1 min. Following 3 days of decolorization with glyceryl lactate, DSE infection and colonization structures were observed under a light microscope. Fungal colonization within the roots was assessed using the slide technique, with 30 random root segments examined microscopically (Biermann and Linderman, 1981). The DSE colonization rate (%) was calculated as the proportion of root segments colonized by DSE.

### 2.4 Isolation of root-colonizing DSE

For DSE isolation, three individual plants of each species were selected at each site. From each plant, eighty 0.5-cm long root segments were excised and sterilized in 75% (v/v) ethanol for 1 min, followed by treatment with 2.5% (v/v) sodium hypochlorite for 2 min. The segments were then rinsed three times in sterile distilled water (Li et al., 2022b). Cultures were incubated on potato dextrose agar (PDA) media supplemented with ampicillin (0.1 g/mL) and streptomycin sulfate (0.1 g/mL) at 27°C in the dark for 5–7 days. Colonies exhibiting dark mycelia were transferred to fresh PDA media for microscopic and macroscopic examination (Maadon et al., 2018).

### 2.5 Molecular identification of DSE

Fresh mycelia (50 mg) of each DSE strain were selected, and DNA was extracted using a DNA kit (Solarbio, China). The reaction system included 1 μL of primer ITS4 (5’-TCCTCCGCTTATTGATATGC-3′), 1 μL of primer ITS5 (5’-GGAAGTAAAAGTCGTAACAAGG-3′), 7 μL of genomic DNA, 11 μL of ddH₂O, and 20 μL of 2 × Es Taq Master Mix. PCR amplification was conducted using a Life ECOTM thermal cycler (BIOER, China) with the following protocol: an initial denaturation at 94°C for 5 min, followed by 35 cycles of denaturation at 94°C for 1 min, primer annealing at 55°C for 1 min, and extension at 72°C for 1 min, concluding with an additional 10-min extension at 72°C. PCR products were then sequenced for analysis. Sequence alignment was performed using MEGA 6.0 software, with BLAST analysis through the National Center for Biotechnology Information (NCBI), and type sequences were selected. A phylogenetic tree was constructed using the maximum likelihood method to identify the taxonomy of the DSE fungi (Tamura et al., 2013; Xie et al., 2017). DNA sequences were submitted to GenBank with accession numbers PP564791, PP564792, PP564793, PP564794, PP564795–PP564810, PP564811, and PP564812. In total, 21 morphologically distinct DSE strains were isolated from 2,160 root segments.

### 2.6 Diversity of the DSE community

Isolation frequency values (IFs) for each isolated DSE strain were calculated by dividing the occurrence of each strain by the total number of fungi isolated. DSE community diversity was assessed using the Shannon-Wiener index (H) and Simpson index (1 − D) (Li et al., 2022b). The evenness index (J) was used to evaluate the uniformity of DSE distribution:


$$H=-\sum \left(P_{i}\right)\left(\ln\;P_{i}\right)$$

$$D=\sum {\left(P_{i}\right)}^{2}$$

where ‘Pi’ is the colonization frequency of each DSE.


$$J=\frac{H}{\ln\left(S\right)}$$

where ‘S’ is the total number of DSE strains.

### 2.7 Heavy metal tolerance of DSE

The screening for heavy metal stress-resistant DSE strains was conducted *in vitro*, and these strains were selected from 22 DSE strains isolated from plants. The experiment utilized modified Melin-Norkrans medium (MMN) with the following composition: glucose 16.0 g; MgSO_4_·7H_2_O 0.15 g; C_5_H_8_O_7_ 0.2 g; (NH_4_)_2_HPO_4_ 0.25 g; CaCl_2_ 0.05 g; NaCl 0.025 g; vitamin B 100 μg; FeCl_3_ 1.2 mL (1%); H_2_O 1,000 mL; Phytagel 9 g; and pH 5.5. The heavy metals selected were Cd and Zn. Cd was applied at concentrations of 0 mg L^−1^ and 40 mg L^−1^ using a CdCl_2_·2.5H_2_O solution, while Zn was tested at concentrations of 0 mg L^−1^ and 1,450 mg L^−1^ using a ZnSO_4_ solution (Zhao et al., 2015).

### 2.8 Illumina sequencing and bioinformatics analysis

Total genomic DNA was extracted from 5 g root samples of *P. australis*, *S. viridis*, and *A. annua via* the E.Z.N.A.® DNA Kit (Omega Biotek, Norcross, GA, U.S.). The DNA quality was assessed *via* 1% agarose gel electrophoresis and a NanoDrop® ND-2000 spectrophotometer (Thermo Scientific Inc.). The upstream primer ITS1 region (ITS1F/ITS2, 5’-CTTGGTCATTTAGAGGAAGTAA-3′ and 5’-GCTGCGTTCTTCATCGATGC-3′) were designed. PCR amplification was performed *via* an ABI GeneAmp® 9,700 PCR thermocycler (ABI, CA, USA). The amplification conditions were as follows: 27 cycles of denaturation at 95°C for 30 s, annealing at 55°C for 30 s, and extension at 72°C for 30 s, followed by a final step of 72°C for 10 min. The PCR products of the duplicate samples were examined *via* 2% agarose gel electrophoresis. The PCR products were then purified *via* the AxyPrep DNA Gel Extraction Kit (Axygen) for gel recovery, and quantified *via* a Quantus™ Fluorometer (Promega, USA). Finally, libraries were constructed *via* the TruSeq™ DNA Sample Prep Kit. The raw sequencing data were submitted to the NCBI Sequence Read Archive (SRA) database. The accession numbers were PRJNA1140829 (*Phragmites australis*), PRJNA1140823 (*Setaria viridis*), and PRJNA1140832 (*Artemisia annua*).

The raw FASTQ files were demultiplexed and quality-filtered using fastp, and sequences were merged with FLASH (Magoc and Salzberg, 2011) based on specific criteria: sequences with ambiguous bases, an average quality score below 20 bp, or lengths shorter than 50 bp were discarded. High-quality sequences were merged based on overlapping regions between read pairs, with mismatches in primer regions removed prior to downstream processing. Low-frequency nonchimeric sequences and singletons were excluded. The optimized sequences were clustered into operational taxonomic units (OTUs) at a 97% similarity threshold using UPARSE 7.1, selecting the most abundant sequence from each OTU as the representative. OTUs were screened manually to remove chloroplast sequences found across all samples, and OTU classification was carried out using the UNITE fungal ITS database^1^. Community composition for each sample was analyzed at various taxonomic levels.

### 2.9 Statistical analysis

Statistical analyses were conducted using IBM SPSS Statistics 26 (SPSS Inc., Chicago, IL, USA). Differences in soil factors, DSE colonization, community composition, and diversity among plant species and plots were evaluated using one-way analysis of variance (ANOVA). The diversity indices of the 22 isolated DSE were calculated using the vegan and ggplot2 packages in R (version 3.3.1). The relative effects of different sites and plant species on soil factors and colonization were assessed *via* two-way ANOVA, with mean values compared through Tukey’s honestly significant difference (HSD) test (*p* < 0.05). Data visualization for soil physicochemical properties and heavy metal contents was performed using Origin 2021 software (Origin Lab, Inc., USA). Correlations between soil physicochemical properties and dominant microorganisms with DSE colonization and isolation frequencies were visualized using the GGally and rstatix packages in R (version 3.3.1) and plotted *via* the ggpairs package.

Alpha diversity indices (Simpson, Chao1, and Shannon) were calculated with mothur software. Venn diagrams were created using the Venn package in R to visually represent shared and unique OTUs across different sites. Bar plots illustrating the composition of highly abundant species in samples were generated with the barplot package in R (version 3.3.1). Co-occurrence network analysis was conducted using 6 R packages (igraph, psych, Hmisc, vegan, dplyr, and reshape2), and microbial co-occurrence networks were constructed in Gephi (version 0.10) to explore plant-fungi co-occurrence relationships (Barberán et al., 2011). Variance partitioning analysis (VPA) was performed using the lattice, pheatmap, and vegan packages in R (version 3.3.1) for statistical analysis and data visualization, examining the effects of plant species, soil properties, and microbial communities on DSE colonization and isolation. A structural equation model (SEM) was applied to analyze the influence of soil factor content, enzyme activity, and fungal community diversity on DSE colonization and isolation, using AMOS 21.0 software.

## 3 Results

### 3.1 DSE colonization status

Hyphae and microsclerotium structures of DSE were observed in the roots of PA, SV, and AA across the three sites (Supplementary Figure S1). The septate hyphae, ranging from brown to dark brown, penetrated both the epidermal and cortical cells of the plant roots (Supplementary Figures S1A–I). Concatenated clusters of microsclerotia were observed filling or colonizing multiple cortical cells (Supplementary Figures S1A–I). DSE colonization rates varied by sampling site and plant species (Supplementary Figure S1J). The highest colonization rates in PA and AA roots occurred at BY, while in SV roots, the highest rate was observed at HD. In both BY and FF, PA roots exhibited the highest DSE colonization rate, whereas in HD, SV roots showed the highest colonization rate.

### 3.2 DSE species composition and diversity

**Figure 1:**
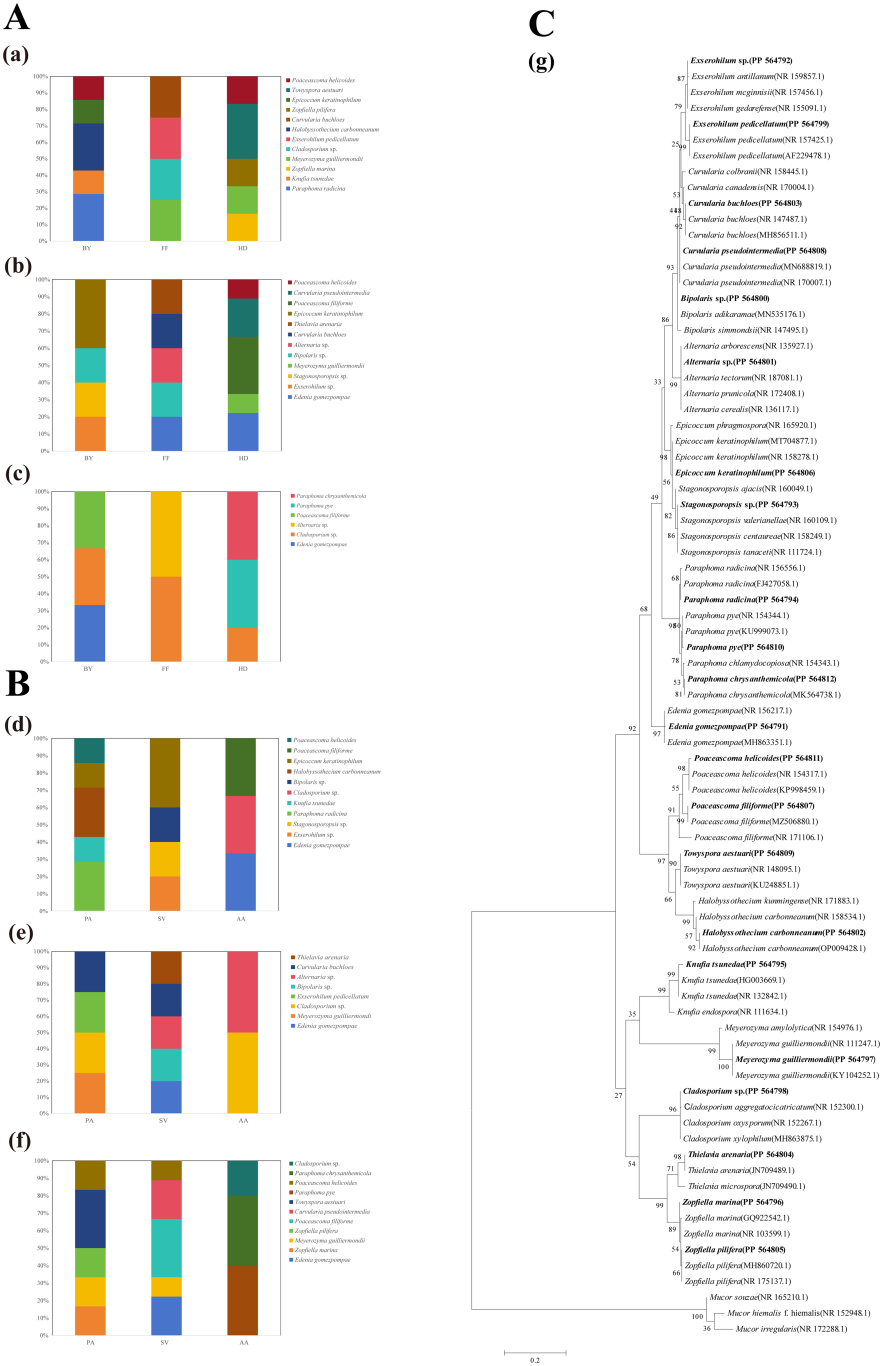
Proportion of endophytic fungal genera in the roots of three plants across three sampling sites **(A,B)** and the maximum likelihood tree of DSE **(C)**. (a) *Phragmites australis*; (b) *Setaria viridis*; (c) *Artemisia annua*; (d) Baiyang Lake; (e) Fengfeng mining site; (f) Huangdao; (g) Maximum likelihood tree generated on the basis of the ITS region sequences of the isolated strains and their closest matches. Each scale bar represents a distance equivalent to 5% of the total nucleotide diversity. The sequences reported in this work are highlighted in bold.

**Figure 2:**
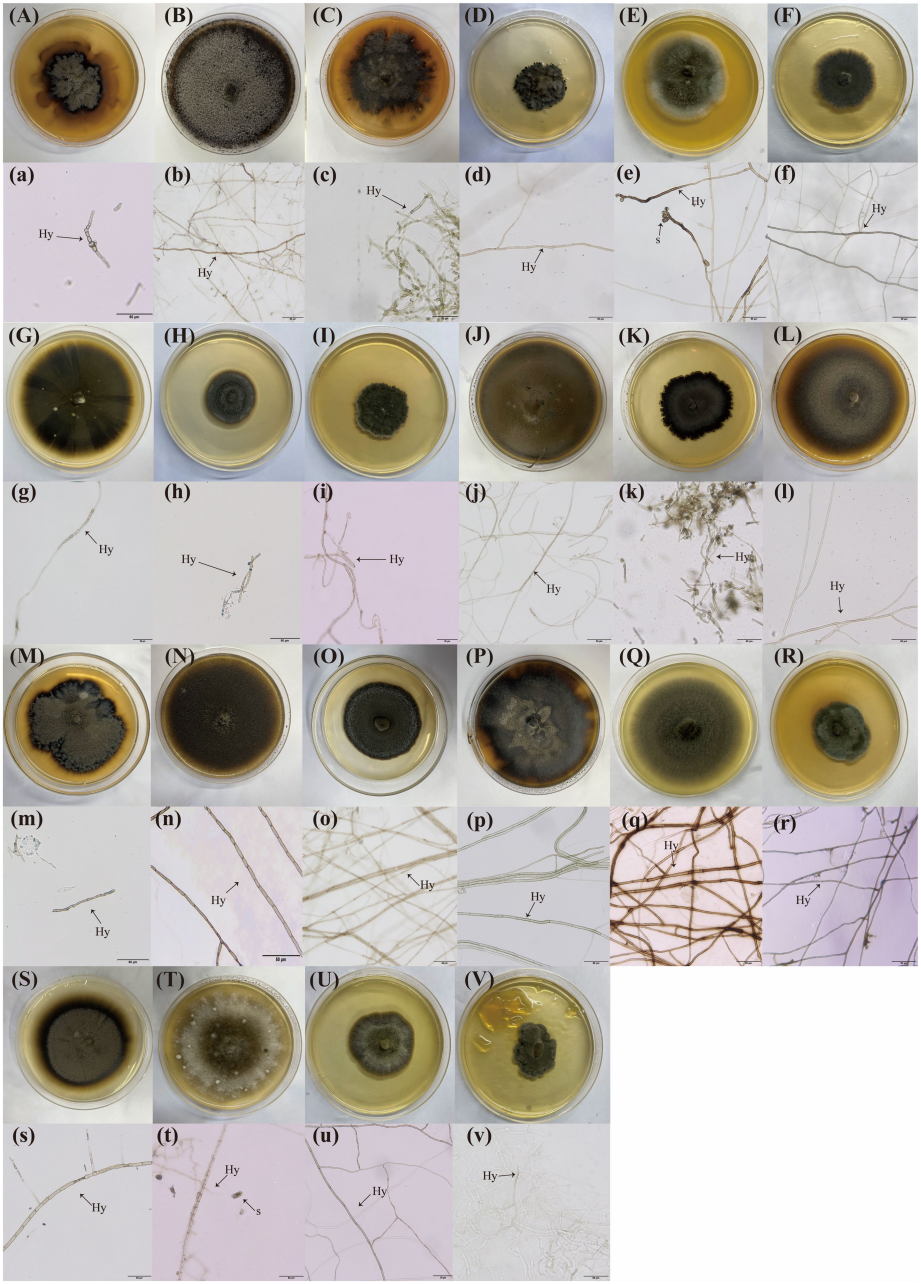
Twenty-two types of dark septate endophytes (DSE) were isolated from the roots of three plant species. **(A–I)** DSE isolated from *Setaria viridis* roots grown on PDA media; **(J–R)** DSE isolated from *Phragmites australis* roots grown on PDA media; **(S–V)** DSE isolated from *Artemisia annua* roots grown on PDA media; (a–v), Microscopic morphology of these DSE isolates. The arrows indicate the following: Hy, DSE hyphae; S, DSE spores.

A total of 84 DSE isolates were obtained and identified through morphological and sequence analysis, representing 16 genera and 22 species (Figure 1C). These were as follows: *Edenia gomezpompae* (*Eg*, Figure 2Aa), *Exserohilum* sp. (*E*sp., Figure 2Bb), *Stagonosporopsis* sp. (*S*sp., Figure 2Cc), *Bipolaris* sp., (*B*sp., Figure 2Dd), *Curvularia buchloes* (*Cb*, Figure 2Ee), *Thielavia arenaria* (*Ta*, Figure 2Ff), *Epicoccum keratinophilum* (*Ek*, Figure 2Gg), *Poaceascoma filiforme* (*Pf*, Figure 2Hh), *Curvularia pseudointermedia* (*Cp*, Figure 2Ii), *Paraphoma radicina* (*Pr*, Figure 2Jj), *Knufia tsunedae* (*Kt*, Figure 2Kk), *Zopfiella marina* (*Zm*, Figure 2Ll), *Meyerozyma guilliermondii* (*Mg*, Figure 2Mm), *Exserohilum pedicellatum* (*Ep*, Figure 2Nn), *Halobysothecium carbonneanum* (*Hc*, Figure 2Oo), *Zopfiella pilifera* (*Zp*, Figure 2Pp), *Towyspora aestuari* (*Tai*, Figure 2Qq), *Poaceascoma helicoides* (*Ph*, Figure 2Rr), *Cladosporium* sp. (*C*sp., Figure 2Ss), *Alternaria* sp. (*A*sp., Figure 2Tt), *Paraphoma pye* (*Pp*, Figure 2Uu), *Paraphoma chrysanthemicola* (*Pc*, Figure 2Vv). Their colonies on PDA were primarily dark brown or black, with a few being gray (Figure 2). The colony appearance was characterized by a felty or wooly texture, with a few colonies exhibiting wavy margins and radial grooves. Microscopic examination of the DSE isolates revealed predominantly dark brown hypha with distinct septa.

The isolation frequencies (IFs) of DSE were analysed on the basis of variations in the sampling sites and host plants (Supplementary Table S1). The results revealed a greater frequency of DSE isolation from BY and HD, with PA and SV showing the highest IFs. Among the sampling sites, *Eg*, *Mg*, *Pf*, and *Ph* presented the highest IFs in HD, whereas *C*sp. had the highest frequency in FF. With respect to the host plants, *Eg*, *Ek*, and *Pf* presented the highest IFs from SV; *Mg* and *Ph* from PA; and *C*sp. from AA (Supplementary Table S1 and Figure 1). In PA, *Mg* was the predominant species, *Pf*, *Ek*, and *Eg* were dominant in SV, AA presented a *C*sp. as the dominant species. *E*sp., *S*sp., *Pr*, *Hc*, *Ek*, and *Kt* were exclusive to BY. *C*sp. was the dominant species in FF, *Eg*, *Mg*, *Pf*, and *Ph* were dominant in HD, with *Zm*, *Zp*, *Cp*, *Tai*, *Pp*, and *Pc* being exclusive to HD (Figures 1A,B).

Sampling sites and plant species had significant effects on DSE diversity, as assessed by the Shannon-Wiener index, Simpson index, and evenness (Supplementary Table S2). Two-way ANOVA results indicated that DSE diversity index values differed significantly among plant species (Supplementary Table S3). Specifically, in BY and HD, PA had the highest Shannon-Wiener and Simpson indices compared to other plants, whereas in FF, SV exhibited the highest values for these indices. Differences in the evenness index across samples were not significant.

### 3.3 Soil physiochemical parameter

**Figure 3:**
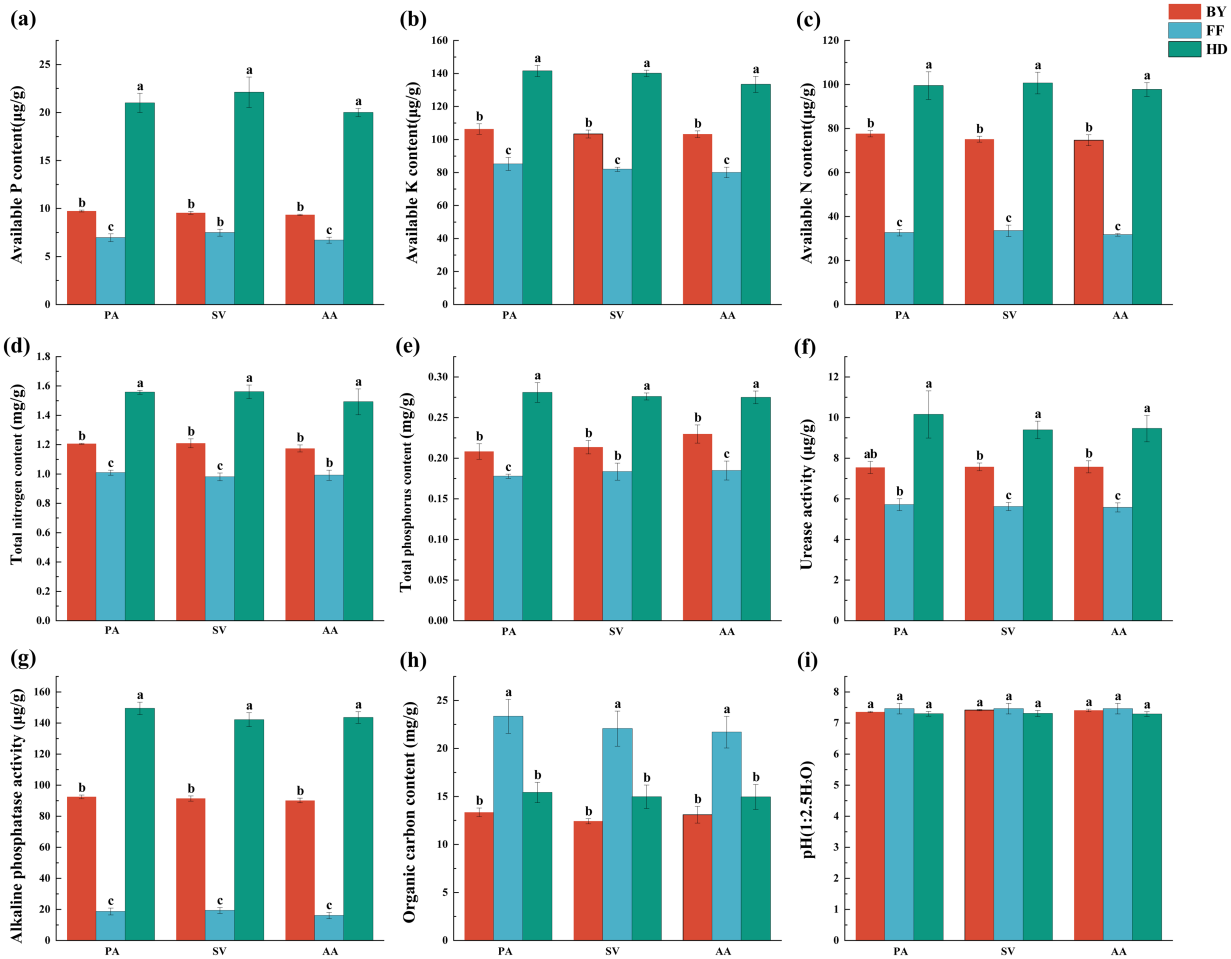
Soil factors contents, and enzyme activities of three plant species across different sampling sites. **(a)** available phosphorus content; **(b)** available potassium content; **(c)** available nitrogen content; **(d)** total nitrogen content; **(e)** total phosphorus content; **(f)** urease activity; **(g)** alkaline phosphatase activity; **(h)** organic carbon content; **(i)** pH. PA, *Phragmites australis*; SV, *Setaria viridis*; AA, *Artemisia annua*; BY, Baiyang Lake; FF, Fengfeng mining site; HD, Haungdao. The differences between the points were analysed by one-way analysis of variance (ANOVA). The error bars represent the standard error (SE). According to the Tukey test, different letters above the error bar indicate significant differences at the *p* < 0.05 level.

**Figure 4:**
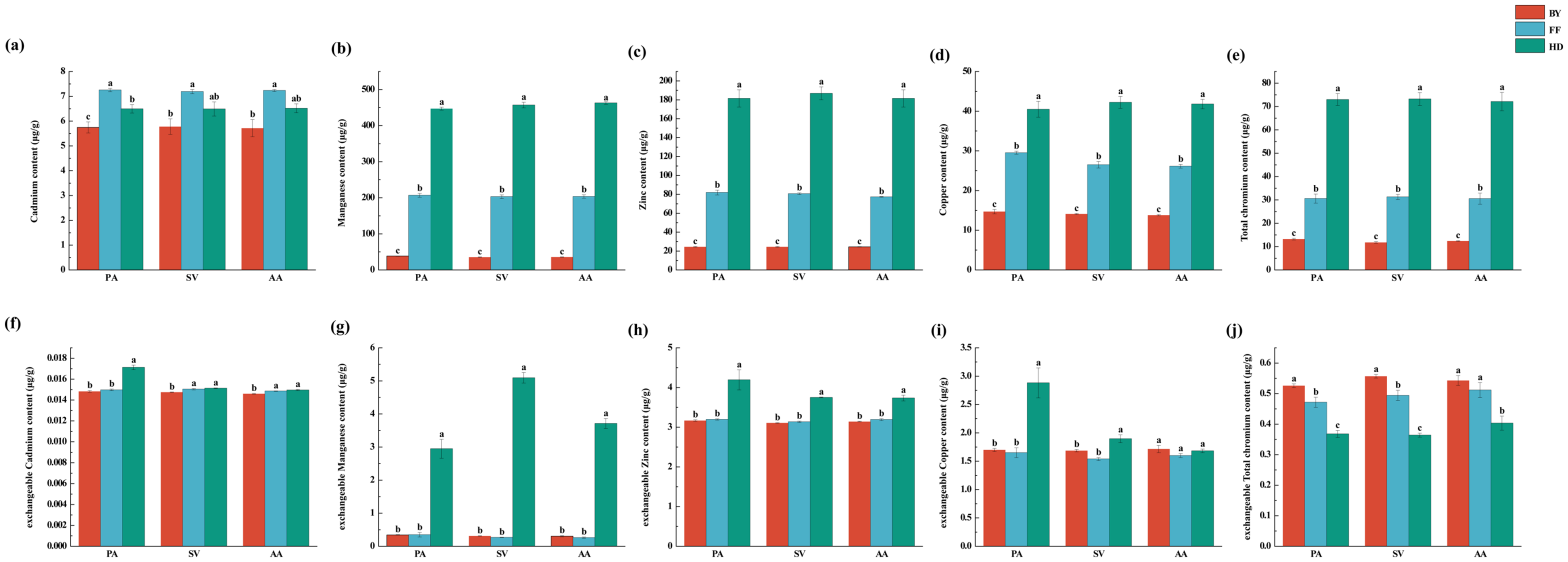
Soil total heavy metal concentrations and exchangeable heavy metal concentrations of three plant species across different sampling sites. **(a)** cadmium content; **(b)** manganese content; **(c)** zinc content; **(d)** copper content; **(e)** total chromium content; **(f)** exchangeable cadmium content; **(g)** exchangeable manganese content; **(h)** exchangeable zinc content; **(i)** exchangeable copper content; **(j)** exchangeable total chromium content. PA, *Phragmites australis*; SV, *Setaria viridis*; AA, *Artemisia annua*; BY, Baiyang Lake; FF, Fengfeng mining site; HD, Haungdao. The differences between the points were analysed by one-way analysis of variance (ANOVA). The error bars represent the standard error (SE). According to the Tukey test, different letters above the error bar indicate significant differences at the *p* < 0.05 level.

Soil factors were significantly influenced by site location, whereas plant species and their interactions had no significant effect on most soil parameters (Supplementary Table S4). Heavy metal concentrations varied significantly among sites. Cd and organic carbon (OC) contents were highest in FF, with the trend FF > HD > BY (Figures 3H, 4A). Additionally, Zn, Mn, Cu, and Cr concentrations were significantly higher at HD than at other sites, following the order HD > FF > BY (Figures 3K–N). Soil TN (total nitrogen), TP (total phosphatase), AP (available phosphatase), AN (available nitrogen), and AK (available potassium) contents, as well as ALP (alkaline phosphatase) and URE (urease) activities, were significantly greater at HD than at the other sites (Figures 3A–G). Among the active heavy metal concentrations in soil, exchangeable Zn, Cu, and Mn reached their highest levels in HD, while exchangeable Cd concentrations for AA and SV were highest at the FF and HD sites. For PA, the maximum exchangeable Cd concentration was observed only in HD. The exchangeable Cr concentration followed an overall trend of BY > FF > HD. Correlation analysis with soil factors revealed a significant positive relationship between soil nutrients, enzyme activity, and the activity of four heavy metals, excluding Cr. In contrast, exchangeable Cr exhibited a significant negative correlation with these factors (Supplementary Figure S2). No significant differences in soil pH were observed across sites, with all sites exhibiting slightly alkaline soils (Figure 3I).

### 3.4 Root-associated fungal community diversity

**Figure 5:**
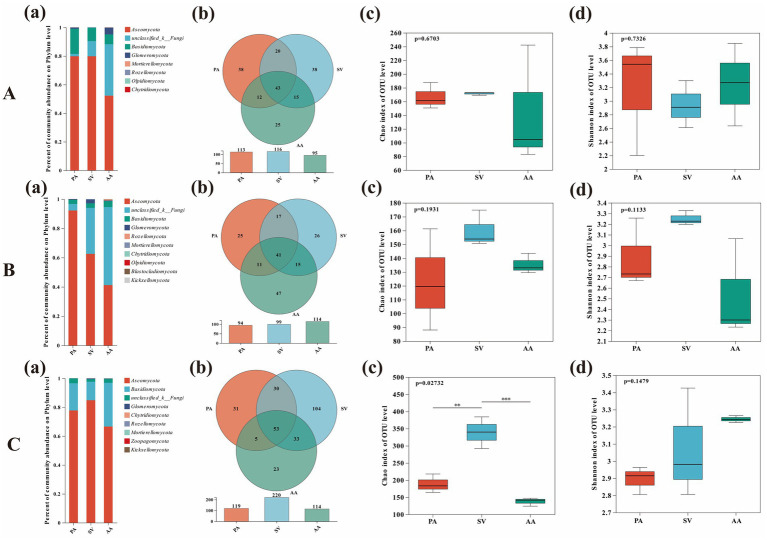
Analysis of fungal diversity, richness, and community composition of three plant species across different sampling sites. **(A)** Baiyang Lake; **(B)** Fengfeng mining site; **(C)** Huangdao. (a) Fungal community composition; (b) Fungal OTUs; (c) Fungal richness; (d) Fungal diversity. PA, *Phragmites australis*; SV, *Setaria viridis*; AA, *Artemisia annua*.

**Figure 6:**
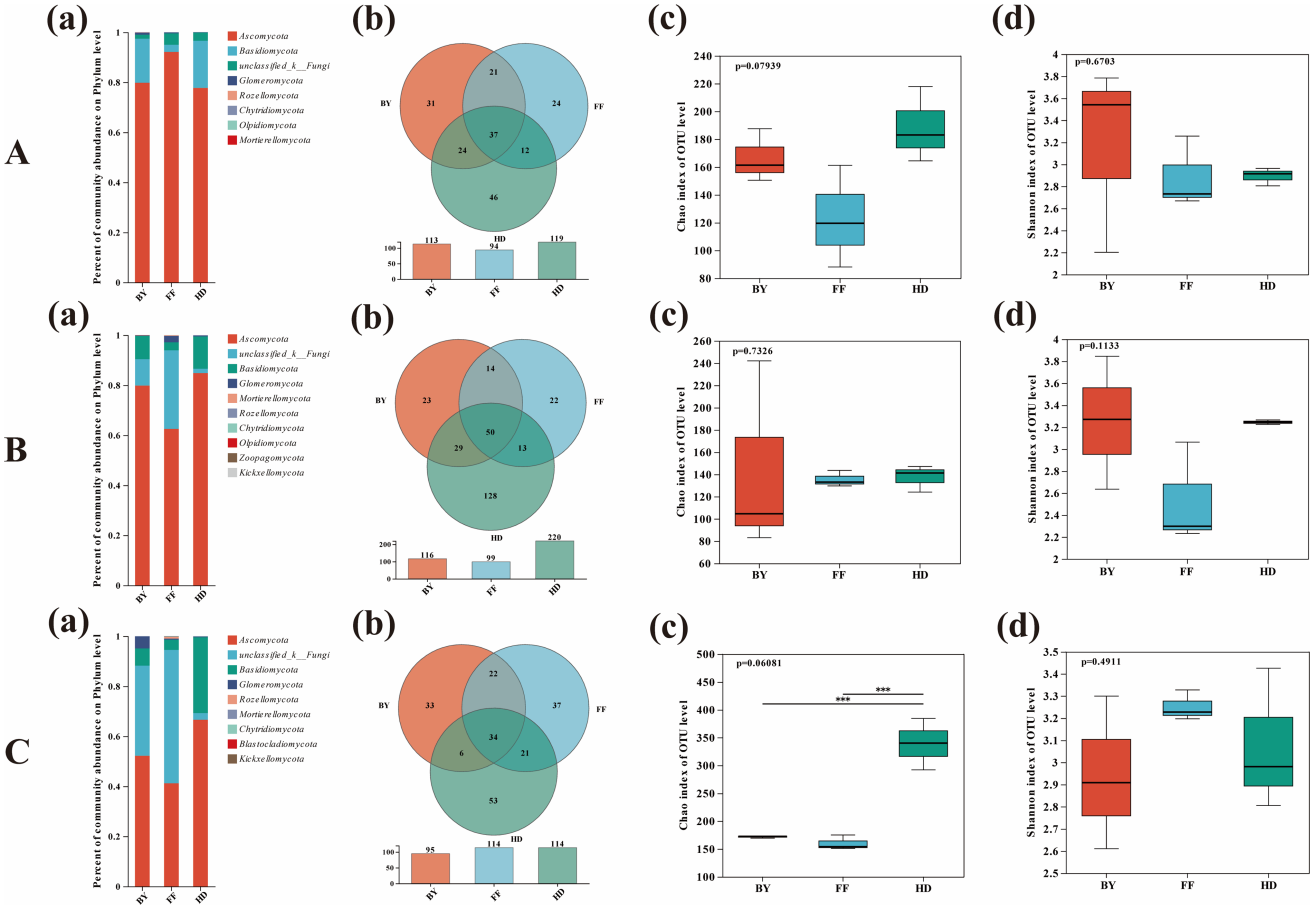
Analysis of the fungal diversity, richness, and community composition of different plant species across the three sampling sites. **(A)** *Phragmites australis*; **(B)** *Setaria viridis*; **(C)** *Artemisia annua*. (a) Fungal community composition; (b) Fungal OTUs; (c) Fungal richness; (d) Fungal diversity. BY, Baiyang Lake; FF, Fengfeng mining site; HD, Huangdao.

The species composition, community structure, and diversity of root-associated fungi showed variable patterns across plant species and sites. At the phylum level, Ascomycota and Basidiomycota were the dominant fungal groups. Notably, there was a higher richness of unclassified fungi in FF compared to other samples, and a greater prevalence of unknown fungal strains in AA compared to other plants (Figures 5Ba,Ca). The Venn diagram showed that the number of site-specific OTUs was highest for PA in BY (38 OTUs), SV in HD (104 OTUs), and AA in FF (47 OTUs) (Figures 5Ab,Bb,Cb). At the host plant level, the OTU count was highest in HD for PA, SV, and AA, with 46, 128, and 53 OTUs, respectively, and lowest in FF, with 24, 22, and 37 OTUs, respectively (Figures 6Ab,Bb,Cb). The Shannon index indicated no significant differences in fungal diversity between plants and sites. However, the Chao1 index showed significantly higher fungal community diversity in SV at HD compared to other plants (*p* < 0.05), with no significant difference between FF and BY (Figure 5Cc). Additionally, fungal community diversity in SV at HD was significantly higher than in other plants (*p* < 0.05), with no significant differences observed among the other plants across sampling sites (Figure 6Cc).

**Figure 7:**
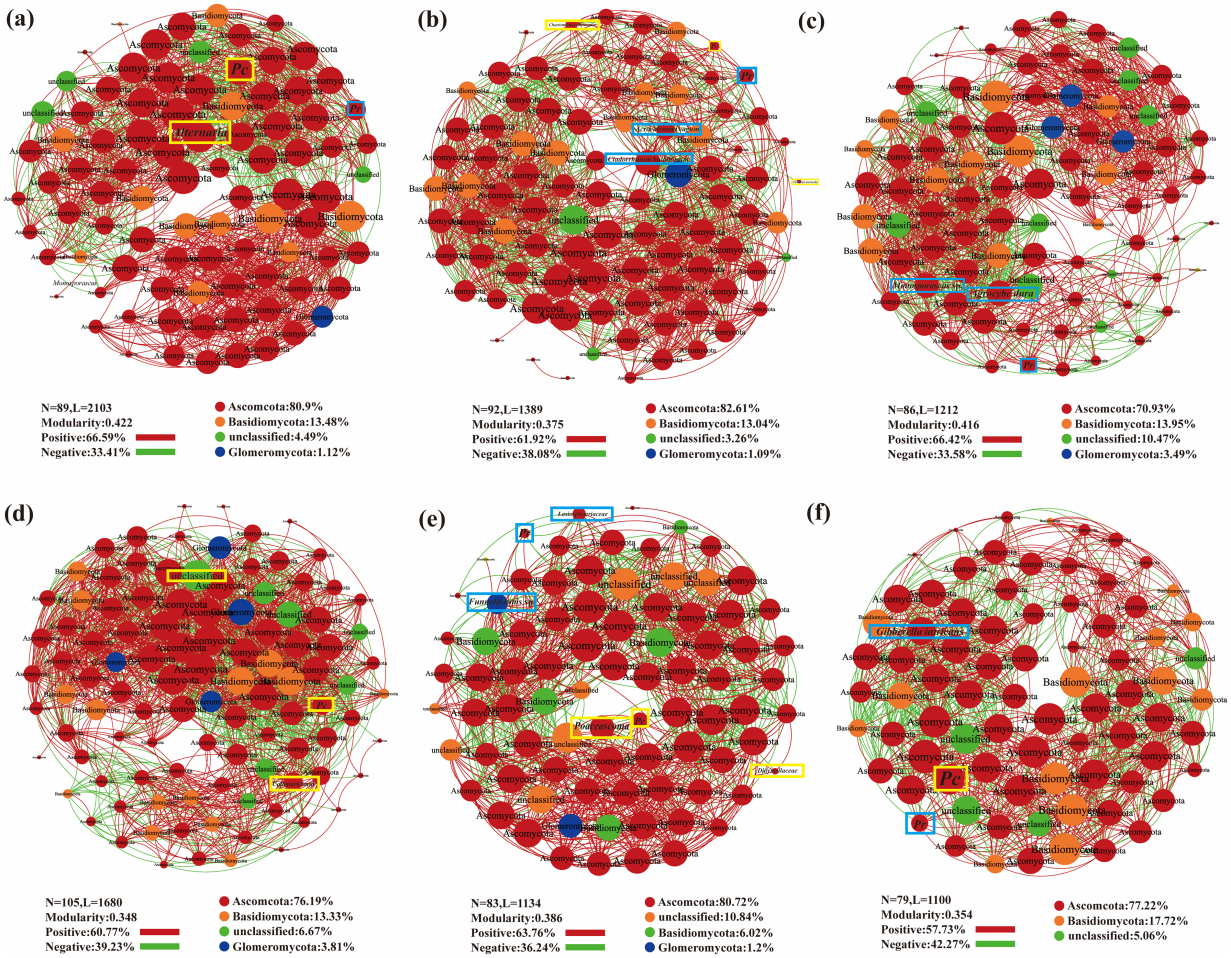
Co-occurrence network plot analysis of three plant species fungal community structure across different sampling sites. **(A)**,*Phragmites australis*; **(B)** *Setaria viridis*; **(C)** *Artemisia annua*; **(D)** Baiyang Lake; **(E)** Fengfeng mining site; **(F)** Huangdao.

Co-occurrence network analysis was performed on the observed OTUs to illustrate the general symbiotic model of root fungi across different sampling sites and plant species (Figure 7). The dominant root fungi, primarily from the Ascomycota and Basidiomycota phyla, were present in all three plants. Across all plant species and sampling sites, the networks were largely dominated by positive associations. Networks for PA and AA had fewer nodes but higher modularity, indicating more stable community structures, with PA exhibiting the highest modularity indices (Figures 7A,B). SV formed the largest network among the three plant species, involving the greatest number of taxa but with the lowest modularity. Among the sampling sites, BY formed the largest network, with a greater number of fungal groups. Additionally, FF exhibited the highest modularity index and the highest frequency of positive interactions within the community, along with a higher node count, suggesting a more stable community structure in FF (Figures 7E,F).

### 3.5 Screening for heavy metal-tolerant DSE

The 22 DSE strains predominantly exhibited brown, dark brown, and grayish-brown colonies in heavy metal tolerance tests on MMN media supplemented with Cd, Zn, or no heavy metal (Supplementary Figure S3). Heavy metal stress affected colony morphology; for example, *E*sp. and *A*sp. appeared grayish-white rather than brown, while *Ek* showed lighter pigmentation and slower growth under Cd stress. Growth diameters were measured to calculate growth rates (Table S5). Under Cd stress, *Eg*, *Ep*, *B*sp., *Cb*, *Tai*, *Ph*, and *Pc* displayed significantly higher growth rates, indicating Cd tolerance. Under Zn stress, *A*sp., *Cp*, and *Pf* showed lighter pigmentation and slower growth, while *Eg*, *E*sp., *Pr*, *Ep*, *Cb*, and *Ta* exhibited significantly higher growth rates, suggesting notable Zn tolerance. Notably, *Eg*, *Ep*, and *Cb* displayed good tolerance to both heavy metals.

Among the heavy metal-tolerant DSE, *Pc* and *Pr* were detected in the amplicon sequence dataset. *Pr* was isolated exclusively from BY and the plant PA, while *Pc* was isolated solely from HD and the plant AA. However, both were detected in plant roots from all three sites through pyrosequencing. The co-occurrence network analysis suggested that *Pc*’s distribution in BY was negatively affected by unclassified fungi and positively influenced by *Colletotrichum*. *Poaceascoma* inhibited *Pc* in FF, while *Didymellaceae* promoted it. The presence of *Pr* in FF was negatively impacted by *Lasiosphaeriaceae* and positively influenced by *Funneliformis* sp. In HD, *Pr* was negatively affected by *Gibberella intricans*, while *Pc* promoted *Pr* presence. In PA, *Alternaria* negatively affected *Pc*, while *Pr* had a positive influence; in SV, *Pc* was negatively impacted by *Preussia terricola*, while *Chaetomium jodhpurense* played a positive role. The presence of *Pr* in SV was negatively influenced by *Acrocalymma vagum* and positively influenced by *Cladorrhinum bulbillosum*. *Monosporascus* sp. inhibited *Pr* in AA, while *Agrocybe dura* promoted *Pr* in AA roots (Figure 7).

**Figure 8:**
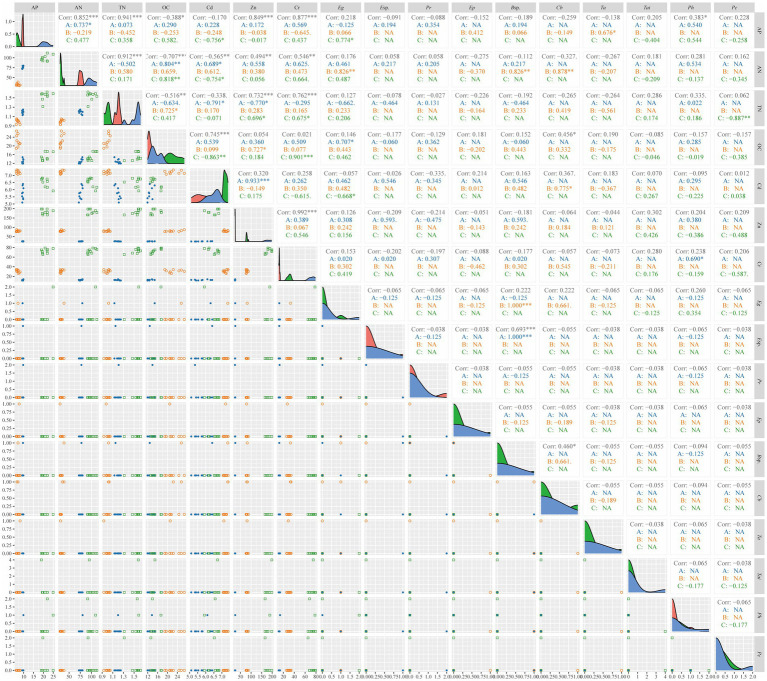
Pair plots of heavy metal-tolerant DSE isolation frequencies with soil factor contents and heavy metal concentrations. Asterisks indicate statistical significance in ggpairs at * < 0.05; ** < 0.01; and *** < 0.001. A, DSE from Baiyang Lake; B, DSE from the Fengfeng mining site; C, DSE from Huangdao.

Soil factors associated with heavy metal sites and the top 15 dominant fungal genera identified by sequencing were selected for correlation analysis with DSE colonization and isolation rates across sampling sites. The analysis revealed that soil environment and the Chao1 index of root-associated fungi had varying impacts on the isolation of heavy metal-tolerant DSE (Figure 8 and Supplementary Figure S4). In BY, increased OC and Cr contents correlated with higher IFs of *Eg* and *Ph*. In FF, increased AP, AN, OC, and Cd contents were associated with higher IFs of *Ta*, *Eg*, *B*sp., and *Cb*, respectively. In HD, increasing AP content was positively correlated with *Eg* IFs, while TN content was significantly negatively correlated with *Pc* IFs (Figure 8). Increasing richness of fungi such as *Sordariomycetes*, *Sarocladium*, *Marasmius*, and *Sordariales* in BY correlated with higher IFs of *E*sp., *B*sp., *Pr*, and *Ph*. In FF, increasing richness of *Sarocladium*, *Poaceascoma*, *Agrocybe*, and *Alternaria* correlated with higher IFs of *Ep*, *Cb*, and *Ta*, respectively. In HD, increased richness of *Agrocybe*, *Sarocladium*, *Fusarium*, and *Paraphoma* corresponded with higher IFs of *Tai*, *Pc*, and *Eg* (Supplementary Figure S4).

### 3.6 Correlation analysis of DSE colonization and isolation rates

**Figure 9:**
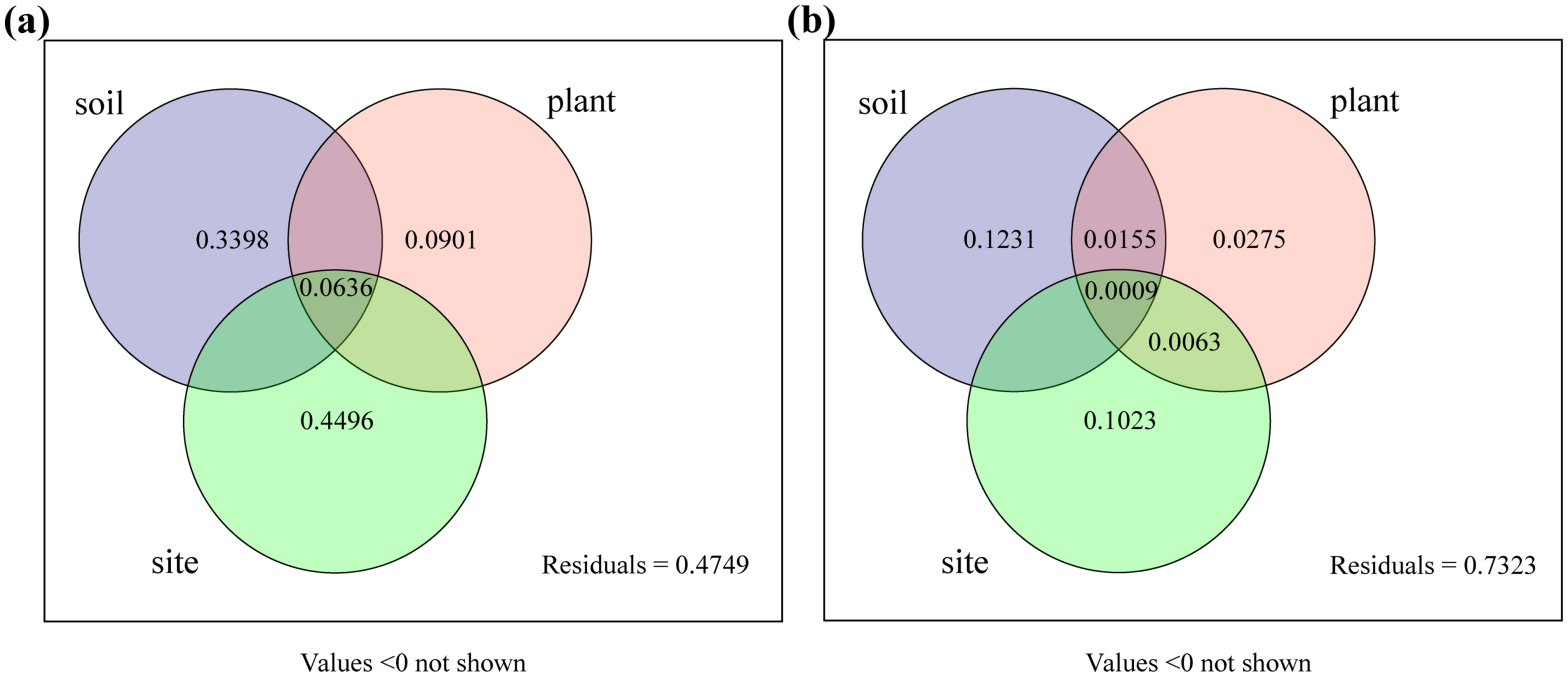
Variation partitioning of DSE colonization and isolation frequencies by plant species, sampling sites, and soil variables. Plant, different plant species; soil, soil variables (including AK, AN, AP, TN, TP, pH, OC, ALP, URE, Cd, and Zn); and sites, different sampling sites. **(A)**, DSE colonization rate; **(B)**, DSE isolation frequency. Values less than 0 are not shown.

Variance partitioning analysis (VPA) was conducted to quantify the contributions of plant species, sampling sites, and soil variables to DSE colonization and isolation rates (Figure 9). Plant species, sampling sites, and soil variables collectively accounted for 94% of the variation in DSE colonization and 28% of the variation in DSE isolation. For DSE colonization rate, soil variables and sampling sites explained 33.98 and 44.96% of the variation, respectively. For DSE isolation rate, they accounted for 12.31 and 10.23% of the variation, respectively. Different plant species contributed 9.01 and 2.75% to DSE colonization and isolation rates, respectively. These findings indicate that sampling sites and soil environments had a greater impact on DSE colonization and isolation than plant species.

**Figure 10:**
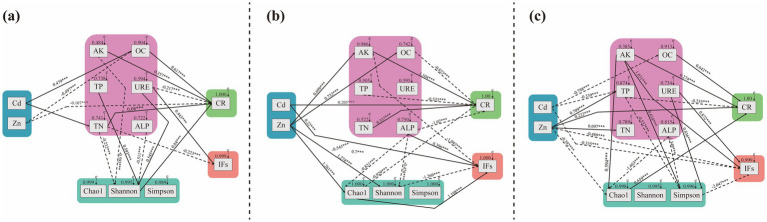
A structural equation model was used to determine the causal relationships among the soil factor contents, enzyme activities, fungal community diversity indices and DSE colonization and isolation. **(A)**, Baiyang Lake; **(B)**, Fengfeng mining site; **(C)**, Huangdao. The solid lines and dotted lines indicate positive correlation and negative correlation pathways, respectively. The width of the solid lines represents the magnitude of the causal impact, whereas the numerical values near the arrows represent the standardized path coefficients (***correlation is statistically significant at a significance level of *p* < 0.001). Cd, soil cadmium content; Zn, soil zinc content; Mn, soil manganese content; Cu, soil copper content; AK, soil available K content; TN, soil total N content; TP, soil total P content; OC, soil organic carbon content; URE, soil urease activity; ALP, soil alkaline phosphatase activity; CR, total colonization rate of DSE; IFs, the isolation rate of DSE; Chao1 index, Fungal richness; Shannon and Simpson indices, Fungal diversity.

Structural equation modeling (SEM) revealed relationships between soil factors, fungal community diversity across different sites, and DSE colonization and isolation rates (Figure 10). In BY, the total DSE colonization rate decreased with increasing Zn concentration and URE activity, while it increased with higher levels of OC, AK, TN, and Shannon index values. The DSE isolation rate in BY increased with rising URE activity but decreased with higher TN content. In FF, total DSE colonization rate positively correlated with increasing Cd concentration, TN, and AK contents, while it decreased with higher OC and TP contents, ALP activity, and Chao1 index values. DSE isolation rate in FF increased with rising Cd and Cu concentrations, ALP activity, and Chao1 index but decreased with higher AK content and Shannon index values, suggesting a positive influence of heavy metal concentrations on DSE distribution in FF. In HD, total DSE colonization rate increased with higher OC and AK contents, Zn concentration, and Chao1 index, while it decreased with rising TP content. The DSE isolation rate in HD increased with higher AK content and URE activity but decreased with higher Cd and Zn concentrations and Simpson index values. Notably, AK content positively influenced DSE colonization across all three sites.

## 4 Discussion

### 4.1 Colonization and diversity of DSE in heavy metal sites

This study demonstrates that environmental conditions and plant species influence the colonization of DSE in heavy metal-contaminated sites, with environmental factors having a greater impact on DSE distribution. Variation in DSE colonization rates across different heavy metal sites highlights their role as key microbial community members within plant roots in these challenging environments (Berthelot et al., 2016; Likar and Regvar, 2013; Xu et al., 2023; Shadmani et al., 2021). The root structure of different host plants can significantly influence endophytic fungal colonization, as plants adapt to external stresses by regulating nutrient transport through their roots (Hou et al., 2019). Our findings indicate that PA and SV exhibited higher DSE colonization rates, likely due to the strong anoxia tolerance and stress resilience of the PA root system, as well as the effective pollutant removal capacity of SV’s fibrous root system (Wang et al., 2024b; Rezania et al., 2019). Environmental changes impact plant growth, subsequently affecting the distribution and colonization of endophytic fungal communities. The high DSE colonization rates observed in BY and HD may result from a synergistic relationship between DSE and their host plants.

The 22 isolated DSE strains belong to the Ascomycota phylum, primarily within the Pleosporales order, consistent with previous reports of DSE presence in heavy metal-contaminated sites (Ban et al., 2017; Narendrula-Kotha and Nkongolo, 2017; Shadmani et al., 2021). DSE often establish mutualistic relationships with their host plants and are frequently isolated from plant roots in heavy metal environments (Farias et al., 2019; Gucwa-Przepióra et al., 2013). Notably, *S*sp., *Kt*, *Hc*, and *Ek* were only found in BY; *Ep*, *Cb*, and *Ta* were specific to FF; and *Zm* and *Zp* were unique to HD. These specific distributions may be influenced by local microbial and soil properties affecting host plant and endophyte activities. Soil organic carbon and oxygen partial pressure are critical factors driving endophytic fungal structure and function. Different endophytic fungal traits support successful colonization, such as the ability to interact with host immune systems, produce growth-promoting hormones, and adhere to host substrates (Zuo et al., 2022). These traits can vary with soil elements and the interactions with surrounding endophytic fungi, contributing to the observed differences in DSE diversity and community composition across environments (Philippot et al., 2023).

The DSE fungi identified in this study are distributed across diverse habitats. For example, *Eg*, *Pp*, *Ta*, and *Cb* have been isolated from desert plants, such as *Haloxylon bunge*, *Corethrodendron scoparium*, and *Gymnocarpos forss* (Li et al., 2024; Zuo et al., 2022). In contrast, *Ph*, *E*sp., *A*sp., *Ep*, *Cb*, and *Pc* have been isolated from plants in heavy metal-contaminated sites, such as *P. australis* and *Astragalus membranaceus* Bunge (Xu et al., 2023; Zhang et al., 2013). In this study, 12 of the 22 identified DSE species are reported in heavy metal habitats for the first time. For instance, *Eg* and *Pr*, previously identified as DSE in desert environments, showed high drought resistance and promoted plant growth under arid conditions (Li et al., 2024; Li et al., 2022a). *Ta*, previously observed in arid soils, has demonstrated both heat resistance and the ability to degrade Bisphenol A (Mtibaà et al., 2018). Our findings indicate that these DSE species, in addition to their known stress-resistance properties, possess heavy metal tolerance, underscoring the ecological significance of DSE for plants thriving in heavy metal-stressed environments.

### 4.2 The effects of soil factors and microbial community on DSE distributions

Our study found that soil organic carbon (OC) and available potassium (AK) were the most significant factors influencing DSE colonization, consistent with previous research (Anand et al., 2023; Chen et al., 2022). Root endophytic fungi, particularly those within the Ascomycota phylum, play an important role in regulating Na-K ion channels and gene expression related to sodium-potassium homeostasis in plants. During symbiosis with the host, these fungi utilize potassium transporters to facilitate the exchange and transport of potassium, effectively mediating soil potassium uptake and enhancing its availability for host plants through close symbiotic networks (Nieves-Cordones et al., 2016; Haro and Benito, 2019). OC serves as the main carbon source for microbial communities, and the rate of organic matter decomposition can influence DSE colonization, the colonization of DSE in the host utilizes soil available C sources to a large extent. The presence of abundant soil TN also contributes to OC content by reducing CO_2_ emissions, thereby supporting DSE colonization in N-rich environments like BY and HD (Han et al., 2021; Guo et al., 2021; Yuan et al., 2022). This explains the observed positive correlation between DSE colonization and OC content in BY and HD, where nitrogen levels are high.

Heavy metals, such as Zn and Cd, disrupt cellular structures and accelerate cell death, creating stressful conditions for microbial communities. Under heavy metal stress, fungal communities shift from energy-consuming anabolism to energy-releasing catabolism. During this process, the growth of fungal communities is suppressed, leading to a decrease in their numbers, which in turn reduces the diversity of root-associated mycorrhizal fungi in the soil (Tang et al., 2021). This aligns with our findings on DSE distribution in BY and HD. In FF, however, heavy metals appeared to positively influence DSE distribution. This does not suggest that Cd and other metals promote fungal growth; rather, it indicates that stable fungal communities in FF possess strong chelation abilities and metal-binding capacities, allowing them to produce extracellular polymeric substances that mitigate heavy metal stress (Luo et al., 2022). The stability observed in FF’s fungal community network, along with DSE heavy metal resistance screening results, supports this hypothesis.

Interactions among fungal species also play a key role in adapting fungal communities to heavy metal pollution, which in turn affects DSE distribution (Liu et al., 2019; Wang et al., 2019). High-throughput sequencing and correlation analysis revealed that, in addition to soil nutrients, the Shannon and Chao1 indices of root endophytic fungi significantly impacted DSE distribution across all sites. Cooperative and competitive interactions among root endophytic fungi enhance biological control by promoting community diversity. These interactions stimulate fungi to release various compounds, activating plant immunity and creating favorable colonization conditions (Matrood et al., 2020; Tao et al., 2023). Fungi from the Basidiomycota and Ascomycota phyla are primary mediators in decomposing plant material due to their hyphal structures and enzyme diversity, which enable them to acquire abundant nutrients that DSE can utilize for colonization. In cases where DSE cannot rely on decomposition, their high stress tolerance and metabolic diversity allow them to maintain community diversity and effectively compete with fungal other communities in nutrient-limited environments (Sun et al., 2021). At the same time, changes in soil conditions further regulate interactions between DSE and their environment, indirectly influencing DSE activity. Elevated P and K levels, along with increased enzyme activity, enhance the availability of soil N and P, which in turn increase root fungal diversity and richness (Shannon and Chao1 indices) (Borase et al., 2020; Teslya et al., 2024). Higher fungal diversity and richness improves the availability of phosphatase transport proteins, boosting metabolic activity, stress resistance, and resilience, thereby facilitating fungal distribution in heavy metal-contaminated sites (Chen et al., 2022; Yu et al., 2024). Sequencing results showed the highest fungal richness in HD and the lowest in FF. This trend was mirrored in soil N and K content and enzyme activity, which explained the higher DSE distribution in HD compared to the lower distribution in FF.

### 4.3 Growth and distribution of heavy metal-tolerant DSE

Among the 22 tested DSE strains, 7 demonstrated strong resistance to Cd stress, while 6 exhibited notable tolerance to Zn stress. Under Cd stress, DSE mycelia were primarily grayish-white or grayish-gray, indicating that Cd significantly inhibited melanin production in DSE compared to both normal conditions and Zn stress (Xu et al., 2023). The majority of these heavy metal-resistant DSE strains were isolated from HD and FF, with fewer from BY. Most DSE isolated from FF displayed high resistance to either Cd or Zn. Correlation analysis of DSE colonization and the distribution of heavy metal-resistant DSE suggests that differences in soil nutrients and fungal community composition across sites likely contributed to this pattern. FF’s higher heavy metal content and stable endophytic fungal community allowed DSE to exchange nutrients with neighboring fungi under high heavy metal stress. Through their strong chelation systems and metabolic capabilities, these DSE could absorb and transfer heavy metal elements, achieving high metal resistance while mitigating stress effects (Tao et al., 2023; Luo et al., 2022).

Higher AN content in FF further supported the distribution of heavy metal-resistant DSE by providing a rich energy source, optimizing their growth conditions, and enhancing metabolic activity (Borase et al., 2020; Yu et al., 2024). The higher richness of endophytic fungi and soil AP content also promoted the distribution of heavy metal-resistant DSE in HD. Abundant endophytic fungi not only provided DSE with a competitive advantage in root colonization and life cycle completion but also facilitated increased hydrolysis of insoluble phosphorus in the rhizosphere, enriching soil P content (Bini et al., 2018; Cheng et al., 2022). Enhanced soil P levels support microbial diversity, increase endophytic fungal activity, and improve DSE distribution in heavy metal sites (Teslya et al., 2024; Xing et al., 2023). These factors collectively influenced the distribution and metal resistance development of DSE.

The distribution of *Pc* and *Pr* varied across sites and was influenced positively or negatively by interactions with other endophytic fungi. Some of these endophytic fungi had been reported, such as *Alternaria*, which often acted as a pathogen causing mycotoxin contamination and consequently inflicted damage on the host, as well as exerted antibacterial effects on the microbial community by limiting the colonization and other life activities of other endophytic fungi at the roots (Somma et al., 2019). Conversely, *Colletotrichum*, although often reported as a pathogen causing anthracnose in various plants, produces bioactive secondary metabolites like sterols, terpenes, and pyrones, which may serve as energy sources for DSE and promote their distribution (Kim and Shim, 2019). Differences in DSE distribution observed in sequencing and isolation experiments support the hypothesis of dynamic interactions among root endophytic fungal communities, highlighting relationships of mutualism and competition (Liu et al., 2019). These interactions are likely key factors influencing DSE colonization and distribution.

## 5 Conclusion

This study identified environmental characteristics of different plots as the primary abiotic factors influencing DSE colonization and distribution. In the three heavy metal sites studied, DSE showed a clear preference for specific plots, and 22 DSE species were isolated. Sequencing results indicated that the richness, diversity, and structural stability of root-associated fungal communities in different plots significantly affected DSE distribution as biological factors. DSE achieved a significantly higher root colonization rates in HD and BY compared to FF, and more DSE isolates were recovered from the both sites. Therefore, we conclude that fungal community diversity and soil nutrients, including soil organic matters and available K contents, actively influence DSE distribution in heavy metal habitats. The community diversity and soil nutrients promoted DSE colonization in nutrient-rich sites such as HD and BY. Furthermore, high heavy metal pollution assembled endophytic fungal communities with stable structure, and cradled DSE strains with higher resistance against heavy metals. Our research on DSE distribution patterns provides more efficient and valuable insights for the future collection of DSE fungal resources, while the findings establish a robust theoretical foundation for exploring and utilizing DSE fungi to mitigate soil heavy metal contamination.

## Data Availability

The datasets presented in this study can be found in online repositories. The names of the repository/repositories and accession number(s) can be found at: https://www.ncbi.nlm.nih.gov/, PRJNA1140829, PRJNA1140823, and PRJNA1140832.

## Footnotes

^1^ https://unite.ut.ee/

## References

[ref1] Alzarhani A. K. Clark D. R. Underwood G. J. C. Ford H. Cotton T. E. A. Dumbrell A. J. (2019). Are drivers of root-associated fungal community structure context specific? ISME J. 13, 1330–1344. doi: 10.1038/s41396-019-0350-y, PMID: 30692628 PMC6474305

[ref2] Anand U. Pal T. Yadav N. Singh V. K. Tripathi V. Choudhary K. K.. (2023). Current scenario and future prospects of Endophytic microbes: promising candidates for abiotic and biotic stress Management for Agricultural and Environmental Sustainability. Microb. Eco. 86, 1455–1486. doi: 10.1007/s00248-023-02190-1, PMID: 36917283 PMC10497456

[ref3] Ban Y. H. Xu Z. Y. Yang Y. R. Zhang H. H. Chen H. Tang M. (2017). Effect of dark septate endophytic fungus Gaeumannomyces cylindrosporus on plant growth, photosynthesis and Pb tolerance of maize (*Zea mays* L.). Pedosphere 27, 283–292. doi: 10.1016/S1002-0160(17)60316-3

[ref4] Barberán A. Bates S. T. Casamayor E. O. Fierer N. (2011). Using network analysis to explore co-occurrence patterns in soil microbial communities. ISME J. 6, 343–351. doi: 10.1038/ismej.2011.119, PMID: 21900968 PMC3260507

[ref5] Becerra A. G. Menoyo E. Faggioli V. Cabello M. Salazar M. J. (2023). Mycorrhizal fungal communities associated with three metal accumulator plants growing in an abandoned Pb smelting factory. Braz. J. Microbiol. 54, 2979–2990. doi: 10.1007/s42770-023-01147-3, PMID: 37864756 PMC10689650

[ref6] Berthelot C. Leyval C. Foulon J. Chalot M. Blaudez D. (2016). Plant growth promotion, metabolite production and metal tolerance of dark septate endophytes isolated from metal-polluted poplar phytomanagement sites. FEMS Microbiol. Ecol. 92:fiw144. doi: 10.1093/femsec/fiw144, PMID: 27364359

[ref7] Biermann B. J. Linderman R. G. (1981). Quantifying vesicular-arbuscular mycorrhizae: a proposed method towards standardization *. New Phytol. 87, 63–67. doi: 10.1111/j.1469-8137.1981.tb01690.x

[ref250] Bini D. dos Santos C. A. da Silva M. C. P. Bonfim J. A. Cardoso E. J. B. N. (2018). Intercropping Acacia mangium stimulates AMF colonization and soil phosphatase activity in Eucalyptus grandis. Sci. Agric. 75, 102–110. doi: 10.1590/1678-992X-2016-0337

[ref8] Borase D. N. Nath C. P. Hazra K. K. Senthilkumar M. Singh S. S. Praharaj C. S.. (2020). Long-term impact of diversified crop rotations and nutrient management practices on soil microbial functions and soil enzymes activity. Ecol. Indic. 114:106322. doi: 10.1016/j.ecolind.2020.106322

[ref9] Chen J. W. Wu Y. Zhuang X. Guo J. J. Hu X. Xiao J. L. (2022). Diversity analysis of leaf endophytic fungi and rhizosphere soil fungi of Korean Epimedium at different growth stages. Environ. Microbiome 17:52. doi: 10.1186/s40793-022-00446-w, PMID: 36271421 PMC9585767

[ref10] Chen S. Zhang G. Q. Liang X. R. Wang L. Li Z. R. He Y. M.. (2023). A dark septate endophyte improves cadmium tolerance of maize by modifying root morphology and promoting cadmium binding to the cell wall and phosphate. J. Fungi 9:531. doi: 10.3390/jof9050531, PMID: 37233243 PMC10219085

[ref500] Cheng X. F. Xie M. M. Li Y. Liu B. Y. Liu C. Y. Wu Q. S.. (2022). Effects of field inoculation with arbuscular mycorrhizal fungi and endophytic fungi on fruit quality and soil properties of Newhall navel orange. Appl. Soil Ecol. 170:104308. doi: 10.1016/j.apsoil.2021.104308, PMID: 34132931

[ref11] Duan Z. B. Luo Y. Wu Y. G. Wang J. Cai X. F. Wen J. C.. (2021). Heavy metals accumulation and risk assessment in a soil-maize (*Zea mays* L.) system around a zinc-smelting area in Southwest China. Environ. Geochem. Health 43, 4875–4889. doi: 10.1007/s10653-021-01003-z, PMID: 34132931

[ref12] Farias G. C. Nunes K. G. Soares M. A. de Siqueira K. A. Lima W. C. Neves A. L. R.. (2019). Dark septate endophytic fungi mitigate the effects of salt stress on cowpea plants. Braz. J. Microbiol. 51, 243–253. doi: 10.1007/s42770-019-00173-4, PMID: 31656023 PMC7058810

[ref13] Gucwa-Przepióra E. Błaszkowski J. Kurtyka R. Małkowski Ł. Małkowski E. (2013). Arbuscular mycorrhiza of *Deschampsia cespitosa* (Poaceae) at different soil depths in highly metal-contaminated site in southern Poland. Acta Soc. Bot. Pol. 82, 251–258. doi: 10.5586/asbp.2013.033

[ref600] Guo Z. M. Zhang X. Y. Dungait J. A. J. Green S. M. Quine T. A. (2021). Contribution of soil microbial necromass to SOC stocks during vegetation recovery in a subtropical karst ecosystem. Sci. Total Environ. 761:143945. doi: 10.1016/j.scitotenv.2020.143945 33360125

[ref14] Han L. Zuo Y. L. He X. L. Hou Y. T. Li M. Li B. K. (2021). Plant identity and soil variables shift the colonization and species composition of dark septate endophytes associated with medicinal plants in a northern farmland in China. Appl. Soil Ecol. 167:104042. doi: 10.1016/j.apsoil.2021.104042

[ref15] Haro R. Benito B. (2019). The role of soil Fungi in K^+^ plant nutrition. Int. J. Mol. Sci. 20:3169. doi: 10.3390/ijms20133169, PMID: 31261721 PMC6651076

[ref16] He Y. Y. Mi B. B. Luo C. Zhao W. J. Zhu Y. L. Chen L.. (2024). Mechanisms insights into cd passivation in soil by lignin biochar: transition from flooding to natural air-drying. J. Hazard. Mater. 472:134565. doi: 10.1016/j.jhazmat.2024.134565, PMID: 38743974

[ref17] Heiri O. Lotter A. F. Lemcke G. (2001). Loss on ignition as a method for estimating organic and carbonate content in sediments: reproducibility and comparability of results. J. Paleolimnol. 25, 101–110. doi: 10.1023/A:1008119611481

[ref18] Hoffmann G. Teicher K. (1961). Ein kolorimetrisches verfahren zur bestimmung der ureaseaktivität in böden. J. Plant Nutr. Soil Sci. 95, 55–63. doi: 10.1002/jpln.19610950107, PMID: 39718201

[ref19] Hou L. F. He X. L. Li X. Wang S. J. Zhao L. L. (2019). Species composition and colonization of dark septate endophytes are affected by host plant species and soil depth in the mu us sandland, Northwest China. Fungal Ecol. 39, 276–284. doi: 10.1016/j.funeco.2019.01.001

[ref20] Hou L. F. Yu J. Zhao L. L. He X. L. (2020). Dark septate endophytes improve the growth and the tolerance of medicago sativa and *Ammopiptanthus mongolicus* under cadmium stress. Front. Microbiol. 10:3061. doi: 10.3389/fmicb.2019.03061, PMID: 32047481 PMC6997539

[ref21] Jeyakumar P. Debnath C. Vijayaraghavan R. Muthuraj M. (2022). Trends in bioremediation of heavy metal contaminations. Environ. Eng. Res. 28:220631. doi: 10.4491/eer.2021.631

[ref22] Jumpponen A. Trappe J. M. (1998). Dark septate endophytes: a review of facultative biotrophic root-colonizing fungi. New Phytol. 140, 295–310. doi: 10.1046/j.1469-8137.1998.00265.x, PMID: 33862835

[ref23] Kim J. W. Shim S. H. (2019). The fungus Colletotrichum as a source for bioactive secondary metabolites. Arch. Pharm. Res. 42, 735–753. doi: 10.1007/s12272-019-01142-z, PMID: 30915681

[ref24] Li M. He C. Wei M. Long J. M. Wang J. R. Yang X. R.. (2024). Temporal and spatial dynamics and functional metabolism of dark septate endophytes of *Gymnocarpos przewalskii* maxim. In Northwest Desert, China. Appl. Soil Ecol. 194:105194. doi: 10.1016/j.apsoil.2023.105194

[ref25] Li X. Liu Y. X. Ye Q. N. Xu M. H. He X. L. (2022a). Application of desert DSEs to nonhost plants: potential to promote growth and alleviate drought stress of wheat seedlings. Agriculture 12:1539. doi: 10.3390/agriculture12101539

[ref26] Li X. Zhang X. Xu M. H. Ye Q. N. Gao H. L. He X. L. (2022b). Improved tolerance of artemisia ordosica to drought stress via dark septate endophyte (DSE) Symbiosis. J. Fungi 8:730. doi: 10.3390/jof8070730, PMID: 35887485 PMC9320036

[ref27] Likar M. Regvar M. (2013). Isolates of dark septate endophytes reduce metal uptake and improve physiology of *Salix caprea* L. Plant Soil 370, 593–604. doi: 10.1007/s11104-013-1656-6

[ref29] Liu H. K. Xu F. Xie Y. L. Wang C. Zhang A. K. Li L. L.. (2018). Effect of modified coconut shell biochar on availability of heavy metals and biochemical characteristics of soil in multiple heavy metals contaminated soil. Sci. Total Environ. 645, 702–709. doi: 10.1016/j.scitotenv.2018.07.115, PMID: 30031328

[ref30] Liu J. Yin M. L. Zhang W. L. Tsang D. C. W. Wei X. D. Zhou Y. T.. (2019). Response of microbial communities and interactions to thallium in contaminated sediments near a pyrite mining area. Environ. Pollut. 248, 916–928. doi: 10.1016/j.envpol.2019.02.089, PMID: 30856507

[ref31] Luo N. Zhang X. J. Chen S. Wang H. X. Liu D. Song J. F. (2022). Effects of cadmium (cd) on fungal richness, diversity, and community structure of haplic cambisols and inference of resistant fungal genera. Environ. Sci. Pollut. Res. Int. 29, 84989–85004. doi: 10.1007/s11356-022-21818-2, PMID: 35788490

[ref32] Maadon S. N. Wakid S. A. Zainudin I. I. Rusli L. S. Zan M. S. M. Hasan N.. (2018). Isolation and identification of endophytic fungi from uitm reserve forest, Negeri Sembilan. Sains Malays. 47, 3025–3030. doi: 10.17576/jsm-2018-4712-12

[ref33] Magoc T. Salzberg S. L. (2011). Flash: fast length adjustment of short reads to improve genome assemblies. Bioinformatics 27, 2957–2963. doi: 10.1093/bioinformatics/btr507, PMID: 21903629 PMC3198573

[ref34] Mateu M. G. Baldwin A. H. Maul J. E. Yarwood S. A. (2020). Dark septate endophyte improves salt tolerance of native and invasive lineages of *Phragmites australis*. ISME J. 14, 1943–1954. doi: 10.1038/s41396-020-0654-y, PMID: 32341473 PMC7367851

[ref35] Matrood A. A. Khrieba M. I. Okon O. G. (2020). Synergistic interaction of Glomus mosseae T. And Trichoderma harzianum R. In the induction of systemic resistance of *Cucumis sativus* L. to *Alternaria alternata* (Fr.) K. Plant Sci. Today 7, 101–108. doi: 10.14719/pst.2020.7.1.629

[ref380] Mtibaà R. Olicón-Hernández D. R. Pozo C. Nasri M. Mechichi T. González J.. (2018). Degradation of bisphenol A and acute toxicity reduction by different thermo-tolerant ascomycete strains isolated from arid soils. Ecotox. Environ. Safe. 156, 87–96. doi: 10.1016/j.ecoenv.2018.02.077, PMID: 29533211

[ref36] Narendrula-Kotha R. Nkongolo K. K. (2017). Microbial response to soil liming of damaged ecosystems revealed by pyrosequencing and phospholipid fatty acid analyses. PLoS One 12:e0168497. doi: 10.1371/journal.pone.0168497, PMID: 28052072 PMC5215397

[ref37] Nieves-Cordones M. Martínez V. Benito B. Rubio F. (2016). Comparison between Arabidopsis and Rice for Main pathways of K^+^ and Na^+^ uptake by roots. Front. Plant Sci. 7:992. doi: 10.3389/fpls.2016.00992, PMID: 27458473 PMC4932104

[ref38] Nong H. J. Liu J. Chen J. Z. Zhao Y. L. Wu L. Tang Y. C.. (2022). Woody plants have the advantages in the phytoremediation process of manganese ore with the help of microorganisms. Sci. Total Environ. 863:160995. doi: 10.1016/j.scitotenv.2022.160995, PMID: 36535473

[ref39] Patel A. K. Singhania R. R. Albarico F. P. J. B. Pandey A. Chen C. Dong C. (2022). Organic wastes bioremediation and its changing prospects. Sci. Total Environ. 824:153889. doi: 10.1016/j.scitotenv.2022.153889, PMID: 35181362

[ref40] Philippot L. Chenu C. Kappler A. Rillig M. C. Fierer N. (2023). The interplay between microbial communities and soil properties. Nat. Rev. Microbiol. 22, 226–239. doi: 10.1038/s41579-023-00980-5, PMID: 37863969

[ref41] Piombo E. Vetukuri R. R. Tzelepis G. Jensen D. F. Karlsson M. Dubey M. (2024). Small RNAs: a new paradigm in fungal-fungal interactions used for biocontrol. Fungal Biol. Rev. 48:100356. doi: 10.1016/j.fbr.2024.100356

[ref42] Rezania S. Park J. Rupani P. F. Darajeh N. Xu X. Shahrokhishahraki R. (2019). Phytoremediation potential and control of *Phragmites australis* as a green phytomass: an overview. Environ. Sci. Pollut. Res. 26, 7428–7441. doi: 10.1007/s11356-019-04300-4, PMID: 30693445

[ref43] Shadmani L. Jamali S. Fatemi A. (2021). Isolation, identification, and characterization of cadmium-tolerant endophytic fungi isolated from barley (*Hordeum vulgare* L.) roots and their role in enhancing phytoremediation. Braz. J. Microbiol. 52, 1097–1106. doi: 10.1007/s42770-021-00493-4, PMID: 33871825 PMC8324717

[ref44] Somma S. Amatulli M. T. Masiello M. Moretti A. Logrieco A. F. (2019). Alternaria species associated to wheat black point identified through a multilocus sequence approach. Int. J. Food Microbiol. 293, 34–43. doi: 10.1016/j.ijfoodmicro.2019.01.001, PMID: 30634069

[ref45] Song S. J. Peng R. S. Wang Y. Cheng X. Niu R. L. Ruan H. (2023). Spatial distribution characteristics and risk assessment of soil heavy metal pollution around typical coal gangue hill located in Fengfeng mining area. Environ. Geochem. Health 45, 7215–7236. doi: 10.1007/s10653-023-01530-x, PMID: 36933105

[ref46] Su Z. Z. Dai M. D. Zhu J. N. Liu X. H. Li L. Zhu X. M.. (2021). Dark septate endophyte Falciphora oryzae-assisted alleviation of cadmium in rice. J. Hazard. Mater. 419:126435. doi: 10.1016/j.jhazmat.2021.126435, PMID: 34323726

[ref47] Sudová R. Kohout P. Rydlová J. Čtvrtlíková M. Suda J. Voříšková J.. (2020). Diverse fungal communities associated with the roots of isoetid plants are structured by host plant identity. Fungal Ecol. 45:100914. doi: 10.1016/j.funeco.2020.100914

[ref48] Sun X. G. Zheng Y. Xu G. Guo Q. Q. Tan J. H. Ding G. J. (2021). Fungal diversity within the phyllosphere of Pinus massoniana and the possible involvement of phyllospheric fungi in litter decomposition. Fungal Biol. 125, 785–795. doi: 10.1016/j.funbio.2021.05.001, PMID: 34537174

[ref49] Tamura K. Stecher G. Peterson D. Filipski A. Kumar S. (2013). MEGA6: molecular evolutionary genetics analysis version 6.0. Mol. Biol. Evol. 30, 2725–2729. doi: 10.1093/molbev/mst197, PMID: 24132122 PMC3840312

[ref50] Tang B. Xu H. P. Song F. M. Ge H. G. Yue S. Y. (2021). Effects of heavy metals on microorganisms and enzymes in soils of lead-zinc tailings pond. Environ. Res. 207:112174. doi: 10.1016/j.envres.2021.112174, PMID: 34637758

[ref51] Tao C. Y. Wang Z. Liu S. S. Lv N. N. Deng X. H. Xiong W.. (2023). Additive fungal interactions drive biocontrol of Fusarium wilt disease. New Phytol. 238, 1198–1214. doi: 10.1111/nph.18793, PMID: 36740577

[ref52] Tarafdar J. C. Marschner H. (1994). Phosphatase activity in the rhizosphere and hyphosphere of VA mycorrhizal wheat supplied with inorganic and organic phosphorus. Soil Biol. Biochem. 26, 387–395. doi: 10.1016/0038-0717(94)90288-7

[ref53] Teslya A. V. Gurina E. V. Poshvina D. V. Stepanov A. A. Iashnikov A. V. Vasilchenko A. S. (2024). Fungal secondary metabolite Gliotoxin enhances enzymatic activity in soils by reshaping their microbiome. Rhizosphere 32:100960. doi: 10.1016/j.rhisph.2024.100960

[ref54] Tripathi A. Awasthi A. Singh S. Sah K. Maji D. Patel V. K.. (2020). Enhancing artemisinin yields through an ecologically functional community of endophytes in *Artemisia annua*. Ind. Crop. Prod. 150:112375. doi: 10.1016/j.indcrop.2020.112375

[ref55] Wang Y. W. Bai D. S. Luo X. G. Zhang Y. (2024a). Effects of *Setaria viridis* on heavy metal enrichment tolerance and bacterial community establishment in high-sulfur coal gangue. Chemosphere 351:141265. doi: 10.1016/j.chemosphere.2024.141265, PMID: 38246497

[ref56] Wang M. Chen S. B. Chen L. Wang D. (2019). Responses of soil microbial communities and their network interactions to saline-alkaline stress in cd-contaminated soils. Environ. Pollut. 252, 1609–1621. doi: 10.1016/j.envpol.2019.06.082, PMID: 31284203

[ref57] Wang Y. H. Feng Z. Wang K. W. Osanyintuyi A. J. (2024b). A new in situ magnetic method to indicate the source and seasonal diffusion of heavy metal contamination at Qingdao Beach, China. Mar. Environ. Res. 198:106516. doi: 10.1016/j.marenvres.2024.106516, PMID: 38678751

[ref58] Wang L. Li Z. R. Zhang G. Q. Liang X. R. Hu L. Y. Li Y.. (2023). Dark septate endophyte Exophiala pisciphila promotes maize growth and alleviates cadmium toxicity. Front. Microbiol. 14:1165131. doi: 10.3389/fmicb.2023.1165131, PMID: 37113231 PMC10126344

[ref59] Xie L. L. He X. L. Wang K. Hou L. F. Sun Q. (2017). Spatial dynamics of dark septate endophytes in the roots and rhizospheres of *Hedysarum scoparium* in Northwest China and the influence of edaphic variables. Fungal Ecol. 26, 135–143. doi: 10.1016/j.funeco.2017.01.007

[ref60] Xing W. L. Gai X. Ju F. Chen G. C. (2023). Microbial communities in tree root-compartment niches under cd and Zn pollution: structure, assembly process and co-occurrence relationship. Sci. Total Environ. 860:160273. doi: 10.1016/j.scitotenv.2022.160273, PMID: 36460109

[ref61] Xu M. H. Li X. Ye Q. N. Gong F. He X. L. (2023). Occurrence of dark septate endophytes in *Phragmites australis* in Baiyang Lake and their resistance to cd stress. Pedosphere 33:9C. doi: 10.1016/j.pedsph.2023.07.009C

[ref62] Xu F. J. Song S. L. Ma C. Y. Zhang W. Sun K. Tang M. J.. (2020). Endophytic fungus improves peanut drought resistance by reassembling the root-dwelling community of arbuscular mycorrhizal fungi. Fungal Ecol. 48:100993. doi: 10.1016/j.funeco.2020.100993

[ref63] Yaashikaa P. R. Kumar P. S. Jeevanantham S. Saravanan R. (2022). A review on bioremediation approach for heavy metal detoxification and accumulation in plants. Environ. Pollut. 301:119035. doi: 10.1016/j.envpol.2022.119035, PMID: 35196562

[ref64] Yan K. Wang H. Z. Lan Z. Zhou J. H. Fu H. Z. Wu L. S.. (2022). Heavy metal pollution in the soil of contaminated sites in China: research status and pollution assessment over the past two decades. J. Clean. Prod. 373:133780. doi: 10.1016/j.jclepro.2022.133780

[ref65] Yu L. H. Zhang Y. F. Wang Y. F. Yao Q. Yang K. J. (2024). Effects of slow-release nitrogen and urea combined application on soil physicochemical properties and fungal community under total straw returning condition. Environ. Res. 252:118758. doi: 10.1016/j.envres.2024.118758, PMID: 38527724

[ref66] Yuan Y. Li J. Yao L. (2022). Soil microbial community and physicochemical properties together drive soil organic carbon in *Cunninghamia lanceolata* plantations of different stand ages. PeerJ 10:e13873. doi: 10.7717/peerj.13873, PMID: 36032943 PMC9406796

[ref67] Zhang W. Q. Dai L. D. Yan Y. G. Han X. Q. Teng Y. J. Li M.. (2024). Multiscale geographically weighted regression-based analysis of vegetation driving factors and mining-induced quantification in the Fengfeng District, China. Ecol. Inform. 80:102506. doi: 10.1016/j.ecoinf.2024.102506, PMID: 39717863

[ref68] Zhang Y. Li T. Zhao Z. W. (2013). Colonization characteristics and composition of dark septate endophytes (DSE) in a lead and zinc slag heap in Southwest China. Soil Sediment Contam. 22, 532–545. doi: 10.1080/15320383.2013.750267

[ref69] Zhao D. K. Li T. Shen M. Wang J. L. Zhao Z. W. (2015). Diverse strategies conferring extreme cadmium (cd) tolerance in the dark septate endophyte (DSE), Exophiala pisciphila: evidence from RNA-seq data. Microbiol. Res. 170, 27–35. doi: 10.1016/j.micres.2014.09.005, PMID: 25294257

[ref70] Zheng F. Guo X. Tang M. Y. Zhu D. Wang H. T. Yang X. R.. (2023). Variation in pollution status, sources, and risks of soil heavy metals in regions with different levels of urbanization. Sci. Total Environ. 866:161355. doi: 10.1016/j.scitotenv.2022.161355, PMID: 36610633

[ref71] Zuo Y. L. Hu Q. N. Liu J. Q. He X. L. (2022). Relationship of root dark septate endophytes and soil factors to plant species and seasonal variation in extremely arid desert in Northwest China. Appl. Soil Ecol. 175:104454. doi: 10.1016/j.apsoil.2022.104454

