# Supplementary material for: Effects of biological and abiotic factors on dark septate endophytes distribution and heavy metal resistance in different environments
Source: Front Microbiol. 2025 Jan 10;15:1527512. doi: 10.3389/fmicb.2024.1527512 (PMC11758167; doi:10.3389/fmicb.2024.1527512)
Supplement: Supplementary file 1 [file Supplementary_file_1.docx]

**Supplementary material**

**Table S1** The isolation rate of fungal species varied among different plant species and sites.

| Strain name | IFs (%) | BY | FF | HD | PA | SV | AA |
| --- | --- | --- | --- | --- | --- | --- | --- |
| *Edenia gomezpompae* | 7.7 | 1 | 1 | 2 | 0 | 3 | 1 |
| *Exserohilum* sp. | 1.9 | 1 | 0 | 0 | 0 | 1 | 0 |
| *Stagonosporopsis* sp. | 1.9 | 1 | 0 | 0 | 0 | 1 | 0 |
| *Paraphoma radicina* | 3.8 | 2 | 0 | 0 | 2 | 0 | 0 |
| *Knufia tsunedae* | 1.9 | 1 | 0 | 0 | 1 | 0 | 0 |
| *Zopfiella marina* | 3.8 | 0 | 0 | 2 | 2 | 0 | 0 |
| *Meyerozyma guilliermondii* | 7.7 | 0 | 1 | 3 | 3 | 1 | 0 |
| *Cladosporium* sp. | 7.7 | 1 | 2 | 1 | 1 | 0 | 3 |
| *Exserohilum pedicellatum* | 1.9 | 0 | 1 | 0 | 1 | 0 | 0 |
| *Bipolaris* sp. | 3.8 | 1 | 1 | 0 | 0 | 2 | 0 |
| *Alternaria* sp. | 3.8 | 0 | 2 | 0 | 0 | 1 | 1 |
| *Halobyssothecium carbonneanum* | 3.8 | 2 | 0 | 0 | 2 | 0 | 0 |
| *Curvularia buchloes* | 3.8 | 0 | 2 | 0 | 1 | 1 | 0 |
| *Thielavia arenaria* | 1.9 | 0 | 1 | 0 | 0 | 1 | 0 |
| *Zopfiella pilifera* | 3.8 | 0 | 0 | 2 | 2 | 0 | 0 |
| *Epicoccum keratinophilum* | 5.8 | 3 | 0 | 0 | 1 | 2 | 0 |
| *Poaceascoma filiforme* | 7.7 | 1 | 0 | 3 | 0 | 3 | 1 |
| *Curvularia pseudointermedia* | 3.8 | 0 | 0 | 2 | 0 | 2 | 0 |
| *Towyspora aestuari* | 7.7 | 0 | 0 | 4 | 4 | 0 | 0 |
| *Paraphoma pye* | 3.8 | 0 | 0 | 2 | 0 | 0 | 2 |
| *Poaceascoma helicoides* | 7.7 | 1 | 0 | 3 | 3 | 1 | 0 |
| *Paraphoma chrysanthemicola* | 3.8 | 0 | 0 | 2 | 0 | 0 | 2 |

Note: BY, Baiyang Lake; FF, Fengfeng mining site; HD, Haungdao; PA, *Phragmites australis*; SV, *Setaria viridis*; AA, *Artemisia annua*.

**Table S2** DSE diversity indexes in roots of three plants in the three sample sites.

| Sites | plants | Shannon-Wiener index | Simpson index | Evenness |
| --- | --- | --- | --- | --- |
|  | PA | 1.562 ± 0.006a | 0.781 ± 0.003a | 0.673 ± 0.003b |
| BY | SV | 1.336 ± 0.003b | 0.722 ± 0.002b | 0.668 ± 0.001b |
|  | AA | 1.096 ± 0.001c | 0.665 ± 0.001c | 0.692 ± 0.001a |
|  | PA | 1.351 ± 0.007b | 0.731 ± 0.004b | 0.675 ± 0.004b |
| FF | SV | 1.606 ± 0.002a | 0.799 ± 0.001a | 0.692 ± 0.001a |
|  | AA | 0.686 ± 0.004c | 0.493 ± 0.004c | 0.686 ± 0.004ab |
|  | PA | 1.566 ± 0.002a | 0.780 ± 0.001a | 0.674 ± 0.001b |
| HD | SV | 1.534 ± 0.005b | 0.770 ± 0.002b | 0.660 ± 0.002c |
|  | AA | 0.691 ± 0.001c | 0.498 ± 0.001c | 0.691 ± 0.001a |

Note: BY, Baiyang Lake; FF, Fengfeng mining site; HD, Huangdao; PA, *Phragmites australis*; SV, *Setaria viridis*; AA, *Artemisia annua*. Means ± standard errors (n = 4). Means followed by the same letter(s) represent significant differences in DSE diversity among different plants under the same sampling sites at *P* < 0.05 according to the Tukey HSD test.

**Table S3** Two-way analysis of DSE diversity indexes in roots of three plants in the three sample sites.

|  | site | | plant | | site*plant | |
| --- | --- | --- | --- | --- | --- | --- |
|  | *F* | *P* | *F* | *P* | *F* | *P* |
| Shannon | 507.01 | **<0.001** | 21341.50 | **<0.001** | 1910.479 | **<0.001** |
| Simpson | 333.46 | **<0.001** | 7094.35 | **<0.001** | 764.44 | **<0.001** |
| Evenness | 8.55 | **<0.001** | 37.92 | **<0.001** | 18.785 | **<0.001** |

Note: Shannon, DSE community diversity index; Simpson, DSE community diversity index, Evenness, DSE species evenness. Bold font indicates significant differences (*P* < 0.05).

**Table S4** Two-way analysis of variance (ANOVA) of soil factors for various sites and different plants.

|  | site | | plant | | site*plant | |
| --- | --- | --- | --- | --- | --- | --- |
|  | *F* | *P* | *F* | *P* | *F* | *P* |
| AP | 366.85 | **<0.001** | 1.74 | 0.203 | 0.56 | 0.694 |
| AK | 7148.53 | **<0.001** | 2.34 | 0.125 | 0.32 | 0.858 |
| AN | 330.81 | **<0.001** | 0.33 | 0.727 | 0.11 | 0.977 |
| TN | 146.81 | **<0.001** | 0.79 | 0.468 | 0.28 | 0.889 |
| TP | 81.84 | **<0.001** | 0.54 | 0.594 | 0.62 | 0.657 |
| pH | 1.63 | 0.223 | 0.04 | 0.964 | 0.03 | 0.998 |
| OC | 45.17 | **<0.001** | 0.44 | 0.653 | 0.11 | 0.979 |
| URE | 46.71 | **<0.001** | 0.28 | 0.762 | 0.21 | 0.929 |
| ALP | 1582.44 | **<0.001** | 1.34 | 0.288 | 0.57 | 0.688 |
| CR | 38.51 | **<0.001** | 32.00 | **<0.001** | 14.88 | **<0.001** |
| Cd | 34.83 | **<0.001** | 0.00 | 0.998 | 0.02 | 0.999 |
| Zn | 788.90 | **<0.001** | 0.25 | 0.780 | 0.18 | 0.944 |
| Mn | 7699.30 | **<0.001** | 0.50 | 0.616 | 1.84 | 0.164 |
| Cu | 556.90 | **<0.001** | 0.76 | 0.484 | 1.87 | 0.159 |
| Cr | 635.905 | **<0.001** | 0.056 | 0.946 | 0.09 | 0.986 |
| ex-Cd | 101.164 | **<0.001** | 70.682 | **<0.001** | 53.455 | **<0.001** |
| ex-Zn | 68.284 | **<0.001** | 3.891 | 0.039 | 2.435 | 0.085 |
| ex-Mn | 870.891 | **<0.001** | 24.154 | **<0.001** | 27.523 | **<0.001** |
| ex-Cu | 26.107 | **<0.001** | 15.311 | **<0.001** | 12.815 | **<0.001** |
| ex-Cr | 86.440 | **<0.001** | 2.905 | 0.081 | 0.825 | 0.526 |

Note: AP, soil available P content; AK, soil available K content; AN, soil available N content; TN, soil total N content; TP, soil total P content; OC, soil organic carbon content; URE, soil urease activity; ALP, soil alkaline phosphatase activity; CR, total colonization rate of DSE; Cd, soil cadmium content; Zn, soil zinc content; Mn, soil manganese content; Cu, soil copper content; Cr, soil total chromium content, ex-Cd, soil exchangeable cadmium content; ex-Zn, soil exchangeable zinc content; ex-Mn, soil exchangeable manganese content; ex-Cu, soil exchangeable copper content; ex-Cr, soil exchangeable total chromium content. Bold font indicates significant differences (*P* < 0.05).

**Table S5** Growth rate of 22 DSE strains under heavy metal stress.

| Strain name | 0 gradient | Zn (1450 mg/kg) | Cd (40 mg/kg) |
| --- | --- | --- | --- |
| *Edenia gomezpompae* | 0.1071±0.0015c | 0.1143±0.0012b | 0.1214±0.0015a |
| *Exserohilum* sp. | 0.3143±0.0038b | 0.4571±0.0026a | 0.2357±0.0018c |
| *Stagonosporopsis* sp. | 0.3643±0.0031a | 0.0000 | 0.1214±0.0012b |
| *Paraphoma radicina* | 0.2357±0.0015b | 0.2429±0.0029a | 0.2286±0.0020c |
| *Knufia tsunedae* | 0.1429±0.0035a | 0.0786±0.0027b | 0.0000 |
| *Zopfiella marina* | 0.0143±0.0010a | 0.0000 | 0.0000 |
| *Meyerozyma guilliermondii* | 0.3929±0.0058a | 0.3000±0.0015c | 0.3857±0.0015b |
| *Cladosporium* sp. | 0.2500±0.0032a | 0.2071±0.0012b | 0.1357±0.0023c |
| *Exserohilum pedicellatum* | 0.1357±0.0015c | 0.1857±0.0025b | 0.2071±0.0021a |
| *Bipolaris* sp. | 0.0786±0.0018c | 0.0429±0.0010b | 0.1500±0.0020a |
| *Alternaria* sp. | 0.3857±0.0019a | 0.2214±0.0029c | 0.2857±0.0015b |
| *Halobyssothecium carbonneanum* | 0.3214±0.0021a | 0.2429±0.0013b | 0.2429±0.0012b |
| *Curvularia buchloes* | 0.2071±0.0015c | 0.2714±0.0026a | 0.2571±0.0012b |
| *Thielavia arenaria* | 0.0286±0.0021b | 0.0929±0.0003a | 0.0071±0.0006c |
| *Zopfiella pilifera* | 0.5643±0.0029a | 0.1714±0.0012c | 0.3571±0.0009b |
| *Epicoccum keratinophilum* | 0.4000±0.0015a | 0.3857±0.0018b | 0.1643±0.0012c |
| *Poaceascoma filiforme* | 0.4571±0.0020a | 0.2571±0.0003c | 0.3714±0.0012b |
| *Curvularia pseudointermedia* | 0.1714±0.0023a | 0.1071±0.0015b | 0.0929±0.0012c |
| *Towyspora aestuari* | 0.3500±0.0012a | 0.2929±0.0017b | 0.3500±0.0028a |
| *Paraphoma pye* | 0.2429±0.0018a | 0.2071±0.0007b | 0.1429±0.0012c |
| *Poaceascoma helicoides* | 0.1429±0.0020a | 0.1214±0.0003b | 0.1429±0.0009a |
| *Paraphoma chrysanthemicola* | 0.1143±0.0017b | 0.1000±0.0009c | 0.1500±0.0009a |

Note: Different letters show significant differences in the growth rate of the same fungal strain under different heavy metal treatments (*P* < 0.05).


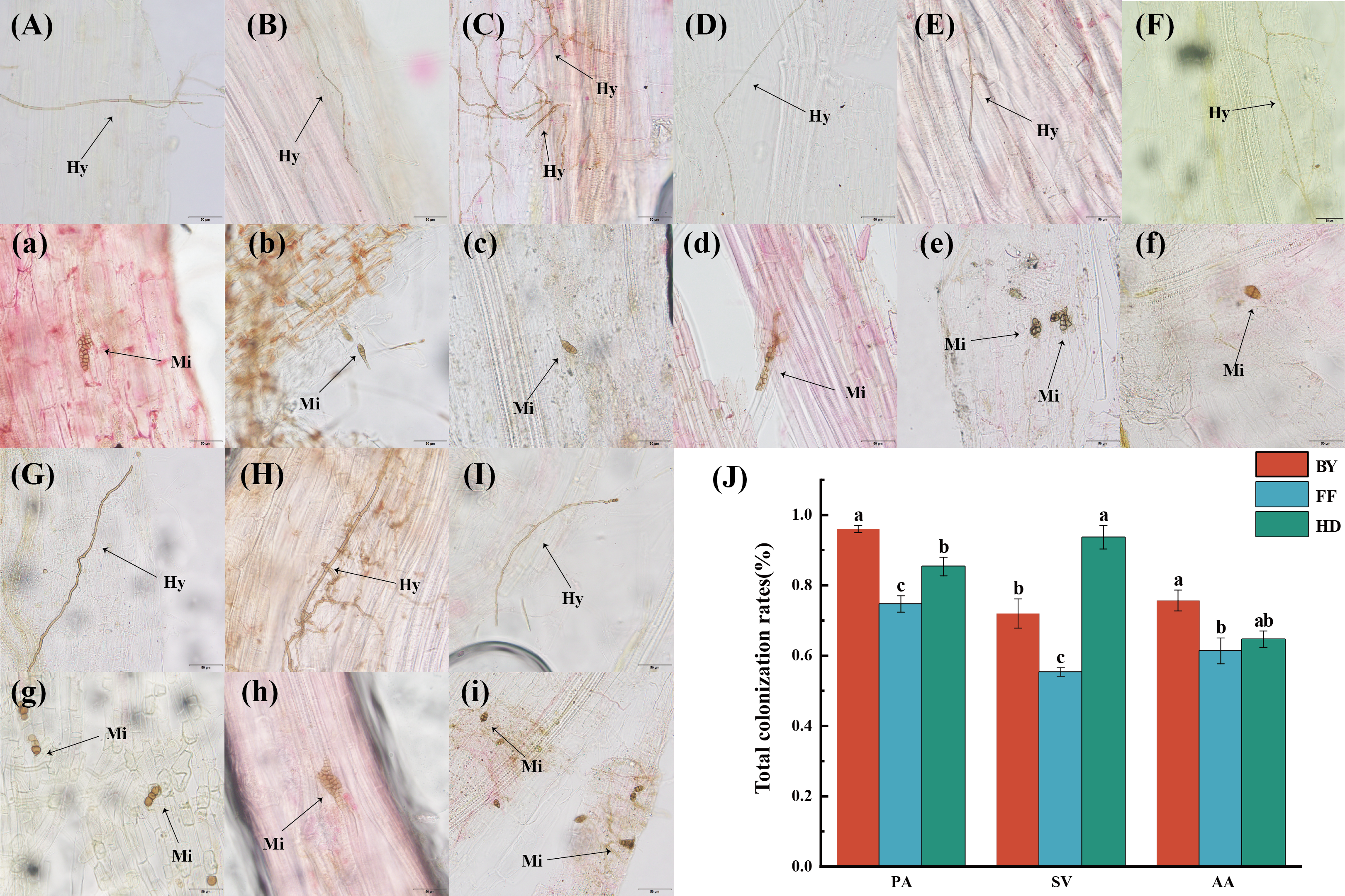


**FIGURE S1.** Colonization status and total colonization rate of dark septate endophytes (DSE) in the roots of three plant species across different sampling sites. The arrows indicate the following: Hy, DSE hyphae. Mi, DSE microsclerotia. (A, D, G. a, d, g), *Phragmites australis*; (B, E, H. b, e, h), *Setaria viridis*; (C, F, I. c, f, i), *Artemisia annua*; (A, B, C. a, b, c), Baiyang Lake (D, E, F. d, e, f), Fengfeng mining site (G, H, I. g, h, i), Huangdao; (J), total colonization rate of DSE. Note: PA, *Phragmites australis*; SV, *Setaria viridis*; AA, *Artemisia annua*; BY, Baiyang Lake; FF, Fengfeng mining site; HD, Haungdao.


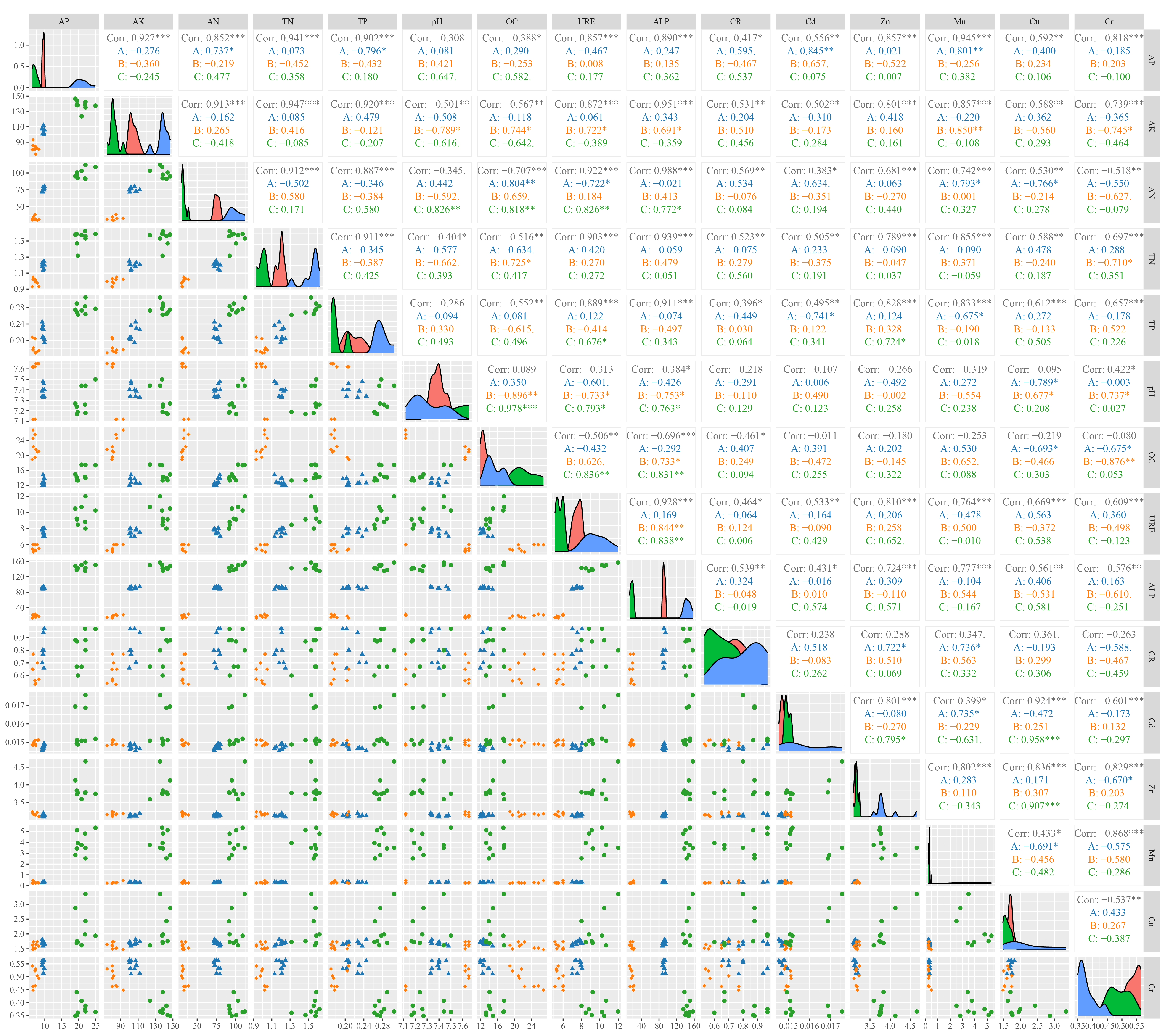


**FIGURE S2.** Pair plots of DSE isolation frequencies and soil factor contents with soil exchangeable heavy metal concentrations. Asterisks indicate statistical significance in ggpairs at * < 0.05; ** < 0.01; and *** < 0.001. A, DSE from Baiyang Lake; B, DSE from the Fengfeng mining site; C, DSE from Huangdao.


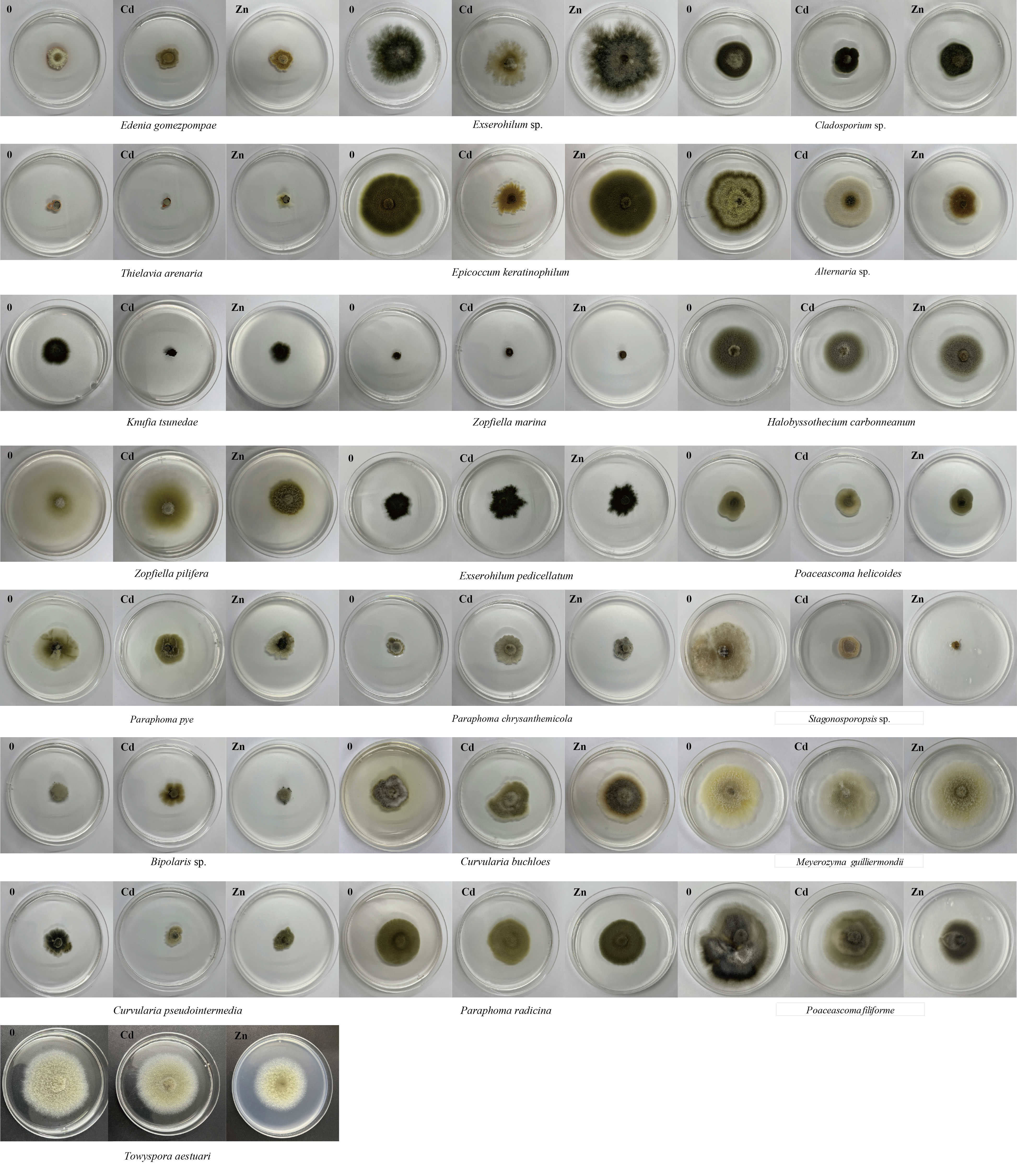


**FIGURE S3.** The colony diameters of DSE grown on MMN solid media at 27 °C were measured. (0), Strains cultured under conventional conditions; (Cd), strains cultured under a Cd concentration of 40 mg/kg; (Zn), strains cultured under a Zn concentration of 1450 mg/kg.


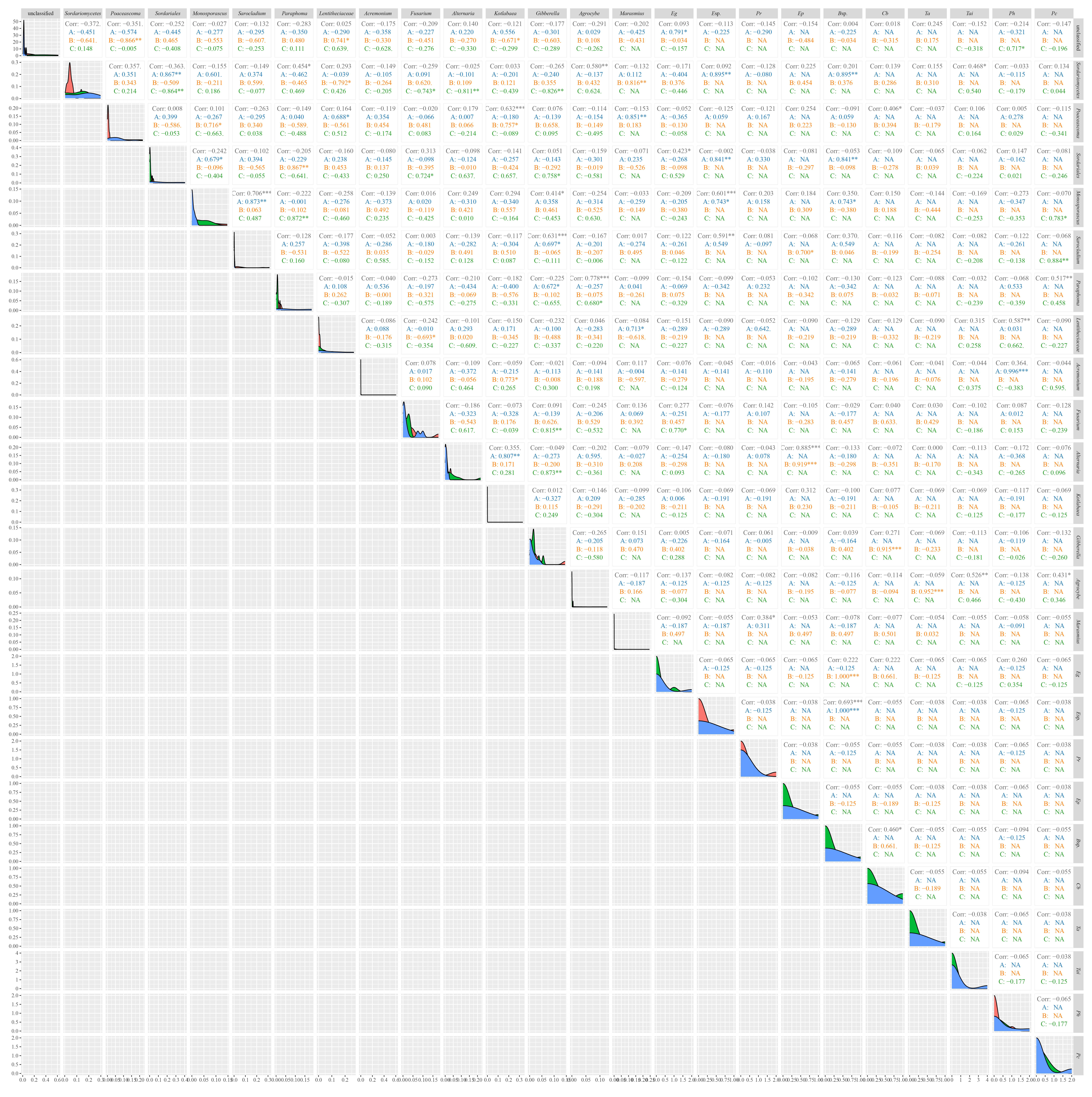


**FIGURE S4.** Pair plots of heavy metal-tolerant DSE isolation frequencies with fungal richness. Asterisks indicate statistical significance in ggpairs at * < 0.05; ** < 0.01; and *** < 0.001. A, DSE from Baiyang Lake; B, DSE from the Fengfeng mining site; C, DSE from Huangdao.

**GitHub link:** https://github.com/dddsx45/ddddsxwzzr.git
